# Discharge deficits in the Central Himalaya linked to greening and warming

**DOI:** 10.1038/s41598-026-68997-3

**Published:** 2026-09-01

**Authors:** Taylor Smith, Bodo Bookhagen

**Affiliations:** Institute of Geosciences, Universität Potsdam, Potsdam, Germany

**Keywords:** Climate sciences, Environmental sciences, Hydrology, Water resources

## Abstract

Anthropogenic climate change and rapid infrastructure development have drastically altered the hydrology of many watersheds, influencing the rate at which water is absorbed, stored, and distributed. In this work, we use long-term daily discharge records in the Central Himalaya alongside rainfall and snowmelt data to explore whether there have been large-scale shifts in the conversion of upstream water into discharge throughout the region. We find that, for the majority of watersheds studied over decadal time scales (*∼ *1960s to 2024), both rainfall and snowmelt are taking longer to be converted into discharge. We link these changes to increasing water storage deficits, where relatively more surface water is being diverted to soil moisture, vegetation uptake, and deeper water storage reservoirs. We propose that large-scale climate change and vegetation greening have modified catchment-level precipitation-discharge relationships in the Central Himalaya.

## Introduction

The Himalaya have seen rapid climate changes in the past few decades, alongside extensive infrastructure development as road networks penetrate deeper into remote areas. Substantial temperature increases have been observed^1^, alongside changes in the cryosphere^2,3^, broad-scale land cover change and vegetation greening^4–7^, and shifting precipitation patterns^8,9^. Changes in precipitation patterns, the timing and volume of snowmelt, and increased water demands from drying soils and more productive vegetation are expected to produce overlapping and non-linear changes in downstream discharge. New infrastructure can also drastically change the speed at which precipitation is converted into downstream discharge, driving both increases (e.g., faster surface runoff on denuded slopes) and decreases (e.g., hydropower dams). Understanding how the relationship between upstream precipitation, snowmelt runoff, and downstream discharge is changing in Himalayan watersheds is of critical importance for successful and sustainable continued development.

A large body of recent work e.g.^10–14^ has noted changes in regional snowmelt patterns, with knock-on influences on discharge and discharge seasonality. In particular, warming does not always lead to faster or earlier snowmelt, and there are large regional differences in the response of discharge to changes in snowpack. Further, the magnitude of snowmelt and discharge changes is often uncertain^15^. There is evidence for a direct increase in discharge due to snowmelt changes in some catchments^7^, but this is not necessarily a universal relationship. In particular, widely reported global vegetation greening^16^, driven by temperature increases and CO$_2$ fertilization^17^, is expected to increase soil-water demands, which, alongside decreasing snowmelt rates in some regions^12^, could change how water propagates downstream. It is thus also possible that discharge may decrease in some catchments even if the snowmelt season intensifies.

Discharge time series encode one part of the water balance of an upstream region. If the inputs (e.g., snowmelt, rain) did not change through time, changes in discharge would be driven by modifications to the characteristics of the watershed itself – for example, infiltration rate, soil-water uptake, irrigation, or slowing of the river due to diversion for hydropower. In the real world, the relationship between discharge and upstream water inputs is more complex, both due to the time-varying nature of inputs (e.g., storm magnitude, snowmelt timing), but also variable levels of water loss due to, e.g., evapotranspiration. For discharge in the Himalaya, the most salient first-order controls to examine are rainfall and snowmelt^8^; changes in the relationship between inputs (rainfall, snowmelt) and outputs (discharge) can hence be used to understand changes in how water moves through the landscape, and what factors control the strength and direction of those relationships.

In this work, we leverage a dense set of discharge measurements across Nepal alongside catchment-summed upstream rainfall, snowmelt, total precipitation, temperature, and evaporation time series from ERA5^18^ and vegetation data from MODIS^19^ to examine potential drivers of changes in downstream discharge. We hypothesize that changes in temperature and precipitation have generally slowed how water is routed through the landscape, with drying soils and greening vegetation absorbing more direct rainfall, increasing soil-water deficits, and leading to the slower release of water into rivers. We contextualize these changes with both climatic shifts and anthropogenic factors such as hydropower projects and road construction.

## Data and methods

### In-situ discharge data

We use a collection of 85 discharge stations that collect daily data from the Department of Hydrology and Meteorology, Nepal, covering the full width of the country, with data spanning from 1962 to 2024 (Supplemental Figure S1, Supplemental Table S1). Data with less than 10 full years are discarded from our analysis, leaving us with 71 long-term stations. For each station, we first flag out any negative values and remove long periods of zero change (e.g., due to frozen rivers), based on having seven consecutive identical values. We fill gaps using linear interpolation up to a maximum of five days in each time series in order to not bias our statistics.

To account for discontinuities in the time series due to, e.g., rating curve changes, we use a quantile matching approach. That is, we first identify breakpoints in the discharge time series using either Bayesian Change-Point Detection and Time-Series Decomposition (BEAST)^20^ or by computing Cumulative Flow Anomalies (CFA) and using piecewise linear segmentation^21^. Using the discovered breakpoints, we then match all discharge segments to a common reference via quantile matching to minimize the influence of step-changes in discharge. We use the raw, BEAST, and CFA discharge values for all analyses, finding that the choice of correction procedure does not generally have an outsized impact on our results. We also compare our results from the corrected and uncorrected discharge data to those found when assessing quickflow, or discharge with the annual baseflow curve removed^22^. We calculate baseflow as the median of 12 different baseflow separation algorithms^22^ in order to apply this approach generally across diverse watersheds in Nepal.

For each station, we calculate the upstream watershed from the discharge station location using standard (D8) flow accumulation routines implemented in TopoToolbox^23^. For those stations with unclear geographic locations (e.g., not directly located on the flow network), we use the closest major stream within 1 km of the provided latitude/longitude of each station. For each discharge time series, we calculate trends in annual mean discharge as well as annual high/low percentiles (5th and 95th) (Supplemental Figures S2–S3). The data all follow similar seasonal distributions (i.e., monsoonal rainfall and dry winters) (Supplemental Figure S4). The watershed outlines used in this study are available on Zenodo^24^, and the environmental characteristics of each watershed can be found in Supplemental Table S2.

### Climate, environmental, and human impact data

For each watershed with more than 10 years of data, we extract daily-aggregated climatic data from ERA5^18^ via Google Earth Engine^25^. We sum (precipitation, snowmelt, evaporation) or average (temperature) the gridded data over each watershed, yielding long-term (1950-2025) daily time series. To contextualize changes in precipitation-discharge relationships, we compute two additional annual-scale metrics from the climate time series: (1) a Simple Daily Intensity Index (SDII, the average precipitation on wet days) and (2) the annual water balance (total precipitation minus evaporation). We further calculate trends in both of these metrics to compare with trends in discharge-precipitation correlation; trends are computed via linear regression, with only statistically significant (*p < 0.05*) trends included in our analysis. We also use MODIS NDVI data (MOD13Q1, 2001–2024^19^) to compute long-term averages and trends in vegetation cover. We further calculate the trend in the annual number of days above the long-term 25th percentile NDVI as a proxy for total growing season length and soil-water uptake intensity.

For comparison, we also include GPM^26^ and CHIRPS^27^ precipitation, as well as GLDAS 2.1^28^ as an alternative model-based climate data set. We note that these data sets have different time periods, so the means and trends from these data are not directly comparable to those based on ERA5; we include these data for reference, but base all analysis on ERA5 data unless explicitly noted. We do not include higher-resolution model outputs e.g.^29,30^ here, as they are generally not available consistently covering all catchments, and hence would make inter-catchment comparisons difficult.

We use five data sets to measure upstream modification of each watershed. (1) Human Footprint Index data (2000–2022)^31^; (2) approved and constructed Nepali hydropower infrastructure projects permitted by the Department of Electricity Development (https://doed.gov.np/pages/powerplantsmorethan1/, compiled April 2025, including only commissioned dams with spatial coordinates); (3) Microsoft Building Footprints^32^ (accessed April 2025) data for Nepal; (4) Open Street Maps tagged road networks (accessed April 2025)^33^; and (5) reported landslides on the Nepali BIPAD portal^34^ (https://bipadportal.gov.np/, compiled April 2025), 1998-2025. Each of these data sets measures a different but overlapping aspect of watershed modification. It is important to note that these data sets are not used to measure the magnitude of impact of each individual change, but rather the general character of each watershed. While it would be useful to know the exact magnitude of each landslide and the year of construction of each building and road, that data is not consistently available. In our analysis, we focus rather on correlations between changes in upstream watersheds and changes in discharge-precipitation relationships. To that end, we reduce the Human Footprint Index data to a simple watershed average and count the number of buildings, roads, infrastructure projects, and landslides per unit area for each watershed. As a final note, building footprints and infrastructure are generally confined to Nepal, while some of the watersheds cross upstream into China; to limit this influence, any watershed with more than 10% of its area in China is removed from comparisons with infrastructure counts.

### Time series correlation

To analyze the correlation between climatic variables and discharge, we first compute Kendall’s *τ * statistics over seasonal (90-day) and inter-annual (3-year) time scales using rolling windows. This allows us to understand generally how discharge responds to different forcings throughout the year, as well as the long-term structure of, e.g., rainfall-discharge correlation. We further compute trends in long-term inter-annual Kendall’s *τ * statistics to understand how the relationship between precipitation and discharge has shifted through time.

As a second step, we use non-negative least-squares to compute the optimal lag time between rainfall and snowmelt and downstream discharge. In short, we generate a set of time-shifted realizations of our input (snowmelt, rainfall) time series from *n*=1–90 days and compute the best-fit non-negative linear model that describes the relationship between input and discharge; we force the fits to be non-negative to maintain the physically realistic constraint that rainfall and snowmelt cannot produce negative discharge. The weighted average of those lag contributions is used here as a metric of transit time, or how long it takes for, e.g., rainfall to be converted into downstream discharge^35^. We compute this metric on a three-year rolling window to assess whether or not transit times have shifted over the course of recent decades. We include an illustrative example for one time series using two different environmental data sources (ERA5 and GLDAS) as Supplemental Figure S5.

We note that snowmelt transit times are much harder to recover, given that rainfall often happens at the same time as snowmelt and overprints the signal of interest. We tested computing both transit times simultaneously by treating the problem as a joint regression; this would allow us to recover rainfall and snowmelt transit times that account for the influence of both variables. We found, however, that this did not improve our results – partitioning snowmelt contributions correctly in monsoonal and intense rainfall catchments remains difficult, and snowmelt transit times should be treated as less reliable than rainfall transit times.

We further note that for all climate comparisons, we subset climate data to the temporal extent of each individual discharge station’s data availability. This means that trends presented for each discharge station do not necessarily cover the same time periods, but any comparison with contextual (e.g., climate) data is done using the same temporal basis as the discharge data. We focus here on showing spatial patterns (using a large number of stations) rather than focusing on the (much smaller) set of stations with several decades of discharge data.

## Results

## Diverging discharge and precipitation trends

**Fig. 1:**
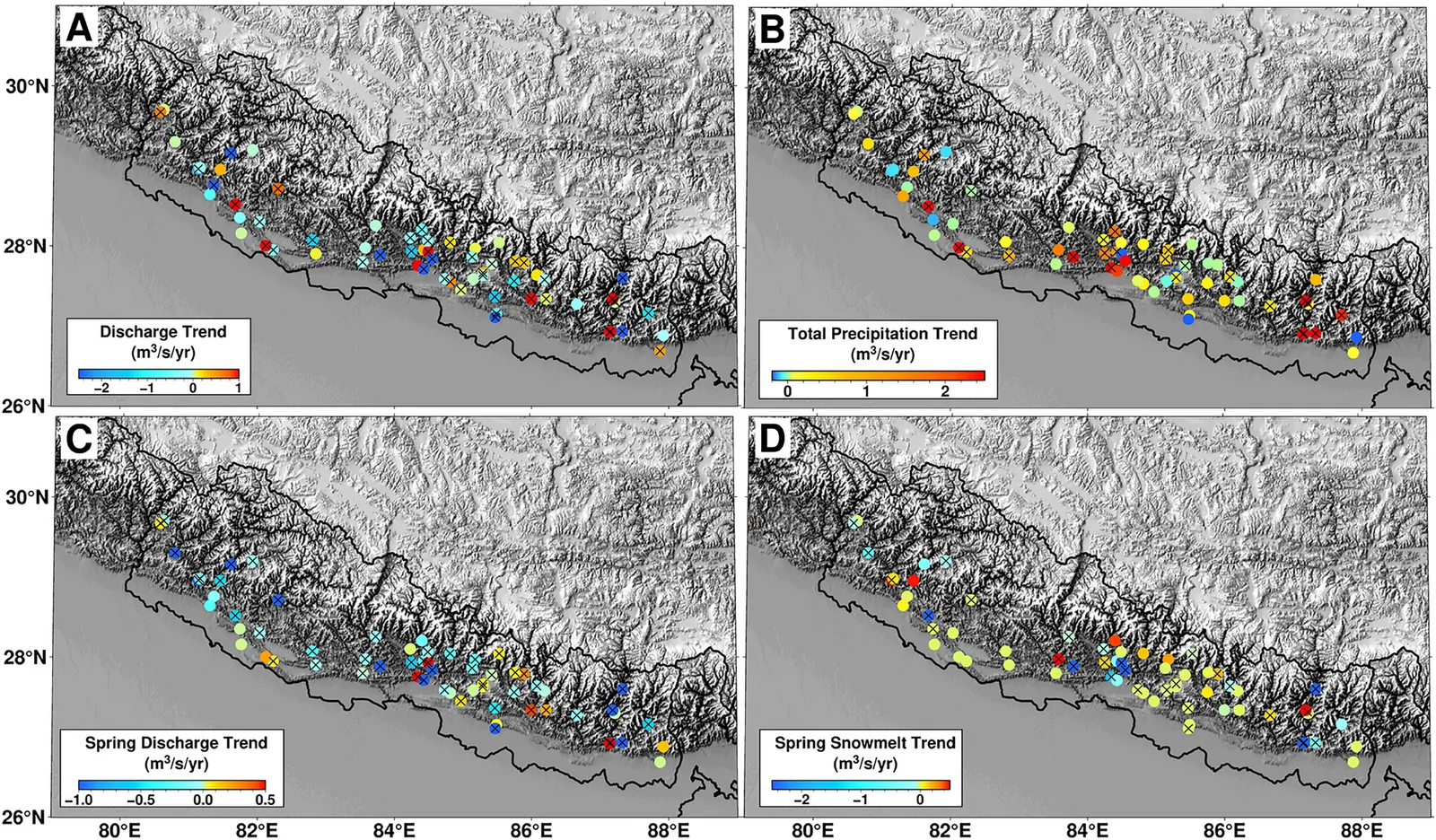
Discharge and precipitation trends. (**A**) Trends in mean annual discharge from in-situ data, and (**B**) trends in mean annual total precipitation from ERA5 data (converted to m$^3$/s equivalent)^18^. Total precipitation trends cover the same period as the discharge trends; see Supplemental Figure S1 for data coverage periods for all stations. (**C**) Same as (**A**), but for the spring (day-of-year 100–150). (**D**) Spring snowmelt trend (from ERA5 data, converted to m$^3$/s equivalent). Discharge trends are generally negative, while total precipitation and spring snowmelt have broadly increased over the same time periods. Statistically significant trends (*p < 0.05*) are marked with black crosses. Maps generated with pygmt (0.17.0)^36^ using the Copernicus DEM as background^37^.

When we consider simple linear trends in both discharge and upstream water (proxied here by total precipitation at the annual scale and snowmelt runoff for the spring season), we find that the trends diverge (Fig. 1). In general, discharge is decreasing across most stations (mean: -0.28 m$^3$/s/year), while upstream water inputs tend to be increasing (mean: 0.95 m$^3$/s/year). We do not see evidence for discharge intensification at substantial-snowmelt stations (defined here as stations that receive at least 25% of their flow from snowmelt, Supplemental Table S2), as has been reported in other parts of HMA^38^ (mean, snowmelt stations: -0.29 m$^3$/s/year). Spring discharge trends are similar to annual-scale trends—despite increasing spring snowmelt in much of our study area—providing further evidence that snowmelt changes are not inducing strong spring discharge intensification in the Central Himalaya; snowmelt-dominated stations, however, have a stronger spring discharge trend (mean, all stations: -0.35; mean, snowmelt stations: -1.09 m$^3$/s/year). Further, if we look at trends in the timing of accumulated discharge (i.e., when 25% or 50% of annual discharge is reached), we find that these thresholds are generally being crossed later in the year for high-elevation and snow-covered catchments (mean, all stations: 0.07; mean, snowmelt stations: 0.14 days/year, Supplemental Figure S6). We posit that this seasonal delay is linked to spring soil-moisture deficits and increased plant-water uptake, which can delay early-season discharge from both rainfall and snowmelt.

### Seasonal correlation structure

**Fig. 2:**
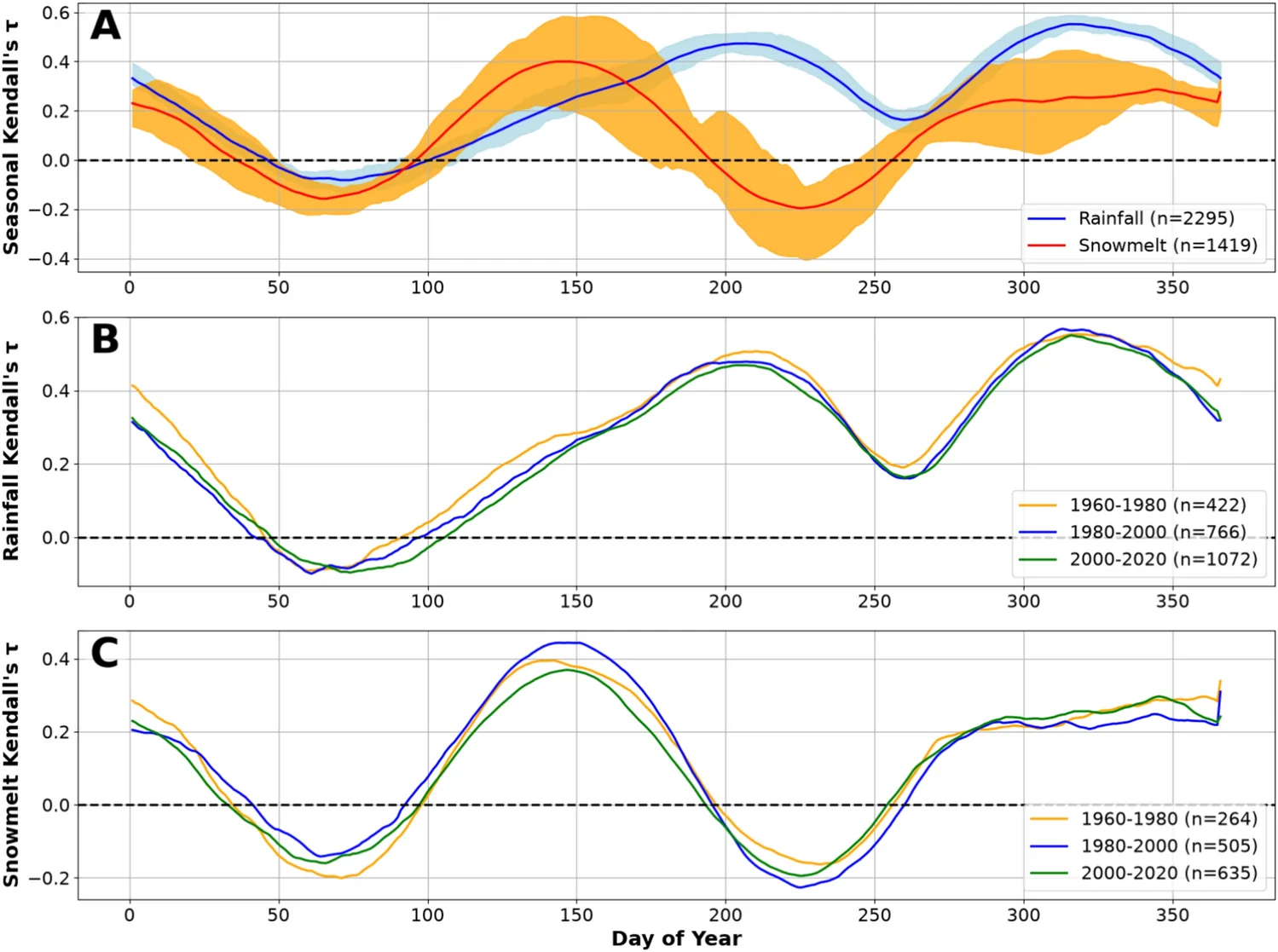
Seasonal correlation. (**A**) Seasonal (90-day rolling) Kendall’s *τ * statistics for rainfall (blue) and snowmelt (orange) correlated with discharge. Averaged by day-of-year and over all watersheds and all station-years; the number of station-years is summed in the legend. Shaded bounds cover one standard deviation. We generally note the most positive correlations during the monsoonal summer season. (**B**) Rainfall and (**C**) snowmelt Kendall’s *τ * split by decade, showing generally similar seasonal correlation structures through time.

The short-term (e.g., last 30, 60, 90 days) relationship between discharge and rainfall or snowmelt will vary throughout the year (Fig. 2). During most of the year, rainfall is positively correlated with discharge; the correlation is weaker during the spring when soils absorb early rainfall and snowmelt contributes significantly to discharge, and in the late monsoon period, when rainfall diminishes, but excess water is still being released from groundwater and saturated soils. This aligns well with previous findings noting that there is significant transient groundwater storage in the Himalaya that buffers stream discharge during the post-monsoon period^39^. The strongest relationship between rainfall and discharge is during the fall season, when plant-water uptake decreases, and smaller rainfall events can be converted directly into discharge over saturated post-monsoon soils. During the spring, snowmelt is more strongly correlated with discharge than rainfall up until the start of the monsoon. Outside of this spring window, it is generally less correlated with discharge; this relationship is enhanced when quickflow (i.e., discharge with baseflow removed) is substituted for raw discharge values in the analysis (Supplemental Figure S7). During the monsoon season, snowmelt-discharge relationships are dominantly negative, indicating that the rainfall signal strongly overprints the influence of snowmelt; that is, low snowmelt can lead to high discharge if there is high rainfall at the same time, hence weakening correlations or even turning them negative.

While some changes in this seasonal relationship were expected, we did not find significant trends in short-term discharge correlation to either rainfall or snowmelt (Fig. 2b,c, Supplemental Figure S7); we note, however, a slight tendency towards lower rainfall-discharge seasonal correlation throughout the year (Fig. 2b). This indicates that the general shape of seasonal correlation of discharge to rainfall and snowmelt has not shifted substantially during our study period.

### Inter-annual correlation

**Fig. 3:**
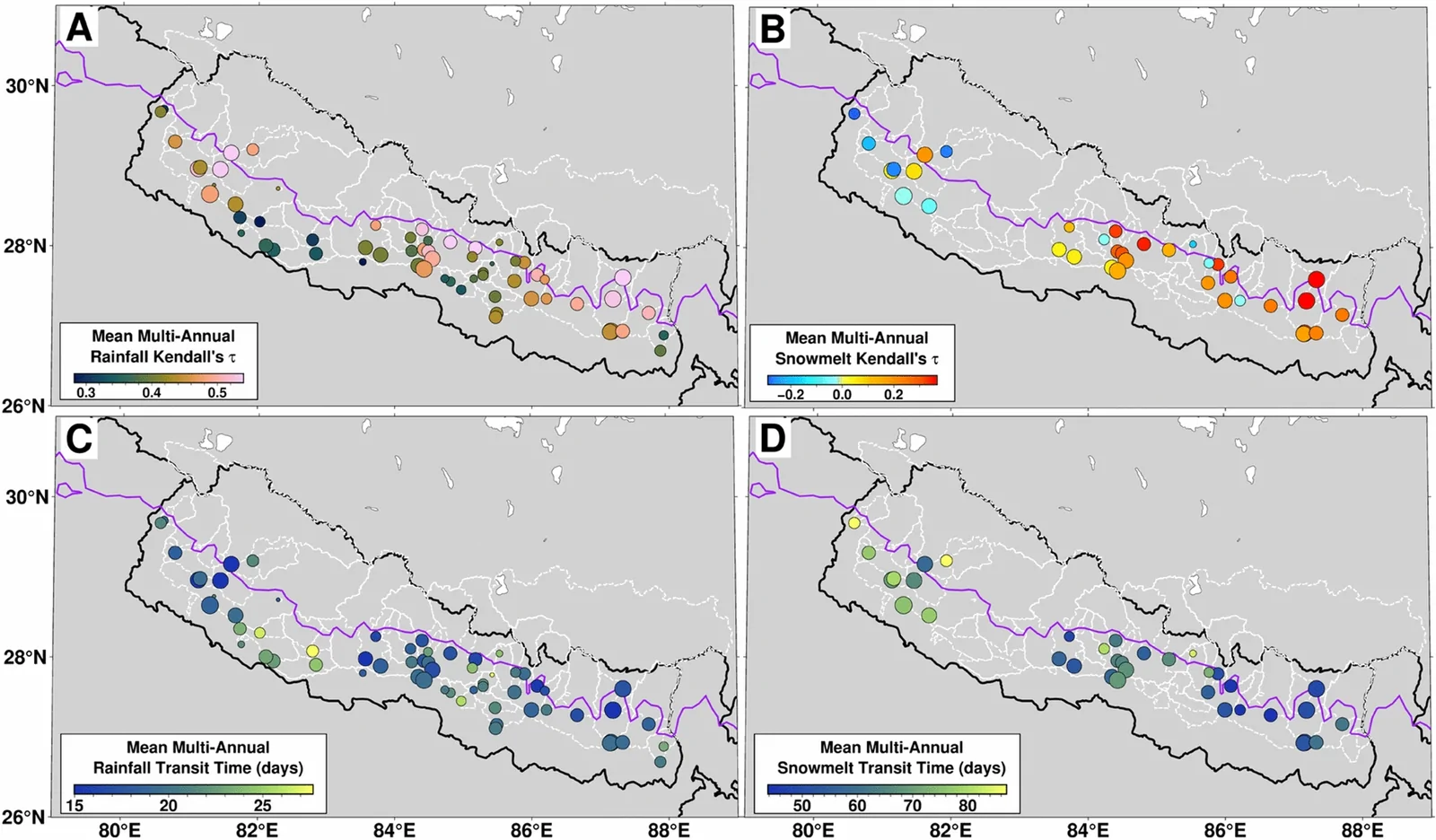
Inter-annual correlation. Kendall’s *τ * averages (three-year window) for (**A**) rainfall and (**B**) snowmelt. Upstream catchment areas shown in white. 2000 m elevation contour shown as purple line. Dots sized by catchment drainage area. Rainfall is positively correlated with discharge, and we generally observe a higher correlation at higher elevations. Average transit times (three-year window) for (**C**) rainfall and (**D**) snowmelt. Rainfall transit times are comparable at medium and high elevations, but lower elevations tend to have longer transit times. Maps generated with pygmt (0.17.0)^36^.

At the inter-annual time scale, Kendall’s *τ * statistics are less influenced by short-term (i.e., seasonal) fluctuations. We find that Kendall’s *τ * statistics for rainfall-discharge are all positive, whereas the relationship between snowmelt and discharge can be both positive and negative (Fig. 3); substituting raw discharge for quickflow increases the Kendall’s *τ * correlation for snowmelt without strongly influencing that for rainfall (Supplemental Figure S8). Using quantile-matched discharge does not influence long-term means (Supplemental Figure S9); and the spatial pattern remains similar when using GLDAS instead of ERA5 climate data (Supplemental Figure S10). High-elevation catchments tend to have stronger rainfall-discharge correlations (mean, all stations: 0.42; mean, stations with mean elevation >2000 m: 0.46); these catchments have relatively less vegetation and shallower soils, which can smooth out the rapid discharge response to rainfall in lower-elevation catchments.

There is an east-west gradient in snowmelt-discharge correlation, with western Nepal being relatively less snowmelt driven. This is also reflected in average transit times, where western Nepal tends to have longer average snowmelt-discharge transit times (Fig. 3d). Snowmelt transit times are almost all longer than rainfall transit times; this behavior is expected due to the longer flow paths from high-elevation snow-water storage to downstream discharge measurement stations.

### Multi-annual changes in correlation

**Fig. 4:**
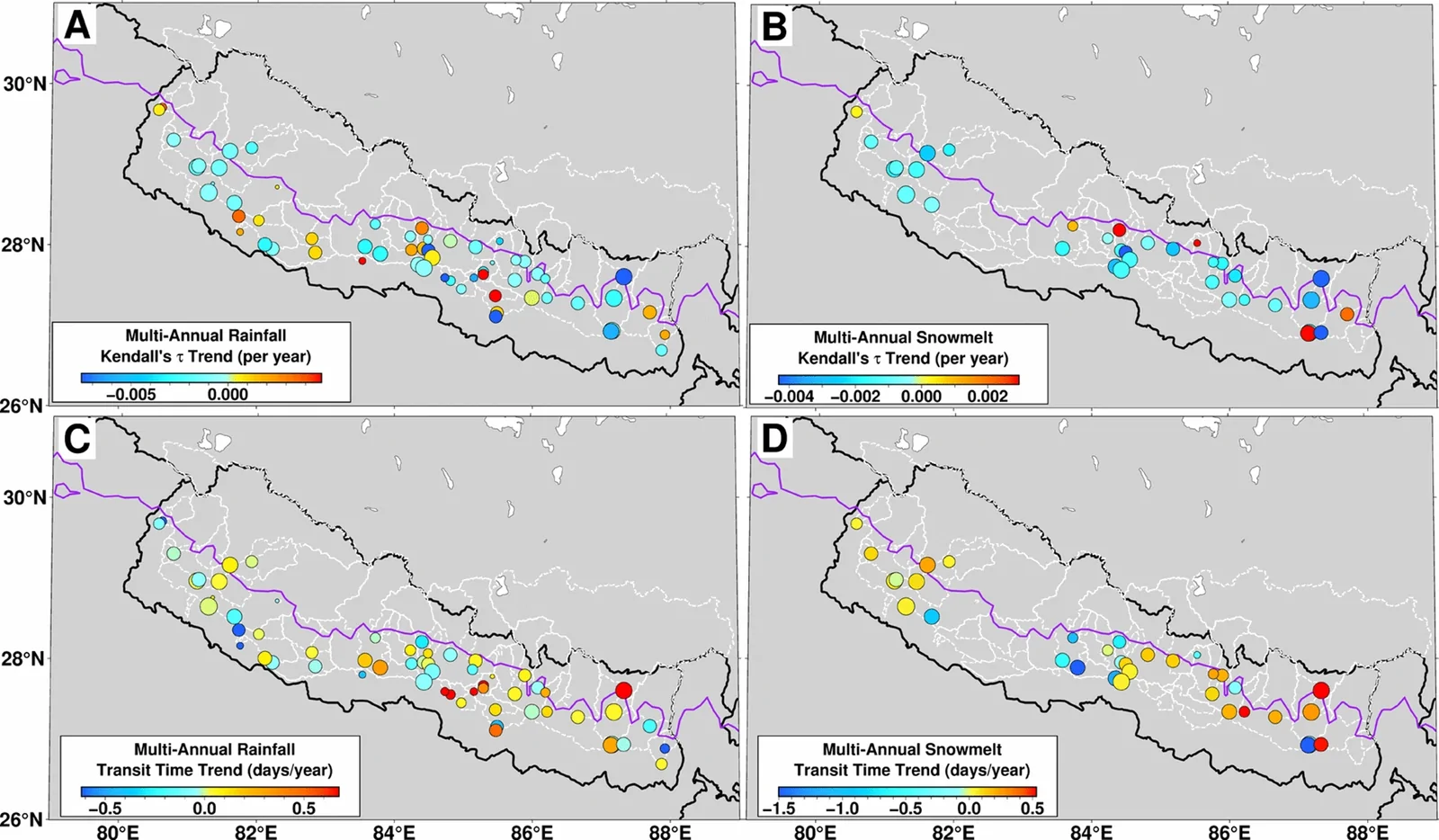
Precipitation-discharge correlation changes. Kendall’s *τ * trend between rainfall and discharge (**A**) and snowmelt and discharge (**B**). Upstream catchment areas shown in white. 2000 m elevation contour shown as purple line. Dots sized by catchment drainage area. Most trends are negative, suggesting a reduction of discharge correlation with both rainfall and snowmelt. Trend in transit time for (**C**) rainfall and (**D**) snowmelt. Trends in transit times for rainfall are generally positive, especially at higher elevations, suggesting that water storage in temporary reservoirs (e.g., soil moisture and groundwater) is increasingly important. Maps generated with pygmt (0.17.0)^36^.

Over longer (multi-annual) time periods, precipitation-discharge correlation averages over seasonal cycles; hence, changes in correlation structure are related to multi-year changes in the relationship between discharge and environmental factors. We can assess these changes station-wise, revealing a distinct spatial pattern in trends in both Kendall’s *τ * statistics and transit times for both rainfall and snowmelt (Fig. 4).

As all correlations are long-term mean positive between discharge and rainfall (Fig. 3a), negative trends in Kendall’s *τ * (Fig. 4a) imply that the majority of discharge stations are becoming less strongly correlated with rainfall; this relationship can also be seen when quantile-corrected discharge is used (Supplemental Figure S11). When quickflow is analyzed instead of raw discharge (Supplemental Figure S12), rainfall Kendall’s *τ * trends are slightly less negative (difference: -3.4 $\times 10^{-4}$), while the other trends remain broadly unchanged. Many stations have seen strong declines in discharge without commensurate negative changes in rainfall (Fig. 1). Transit times for rainfall are generally increasing (median: 0.05 days/year); this would be expected in the case of increased transient rainfall storage or absorption by, e.g., vegetation.

Discharge-snowmelt correlations are on average decreasing (median: -0.01), with stronger increases in transit time than those found between rainfall and discharge (median: 0.07 days/year). However, there are also many catchments with decreasing transit times, indicating that in some cases, snowmelt is being more rapidly converted into downstream discharge. This could happen due to snowmelt intensification during the spring, or also due to shifts in the timing of snowmelt. Those same timing shifts could also increase transit times and decrease correlation, if, for example, snow is pushed to higher elevations, resulting in later melt (e.g., during the monsoon) and longer transit from high-elevation storage to low-elevation discharge measurement stations.

## Discussion

Previous work has found both positive and negative discharge trends throughout Nepal, depending on the size and location of the catchment^40–44^. We find a slight increase in annual catchment-aggregated total precipitation throughout Nepal using ERA5 data, alongside broadly decreasing annual average and spring discharge during the same time periods (Fig. 1). We find statistically significant trends in rainfall- and snowmelt-discharge correlation, indicating multi-annual scale changes in the hydrological cycle of Nepal (Fig. 4). There are many processes that can influence the rate at which upstream water inputs are converted into discharge, and hence the strength of the linear correlation and optimal lag time between watershed inputs and outputs. These trends are broadly similar across discharge bias correction methods (Supplemental Figure S13) and when comparing different upstream input data (Supplemental Figure S14).

Anthropogenic influences are one candidate driver; hydropower infrastructure and other dams have a particularly strong influence on how quickly water moves downstream. Changes in land use (e.g., replacing forests with agriculture) and other types of infrastructure—particularly roads—can also strongly impact watershed hydrology^42,45,46^. If we consider both anthropogenic changes (roads, buildings, land cover) and natural structural changes (landslides) alongside trends in rainfall-discharge correlation, we do not find statistically significant correlation with Kendall’s *τ * trends (Supplemental Figure S15). However, we lack information on the timing of landscape modifications, which limits our ability to directly link changing human land-use to rainfall-discharge correlation changes. Further, the scale of the human-influence data is generally a poor match for our analysis—spatially consistent data sets covering the whole study area are rare, meaning that some of our analysis is based upon lower-resolution regional data. It is possible that more concise relationships between anthropogenic land-use change and precipitation-discharge changes would be apparent in the analysis of individual catchments, particularly if high-quality hydropower, landslide, building footprint, and road network data were collected over a smaller focused area.

**Fig. 5:**
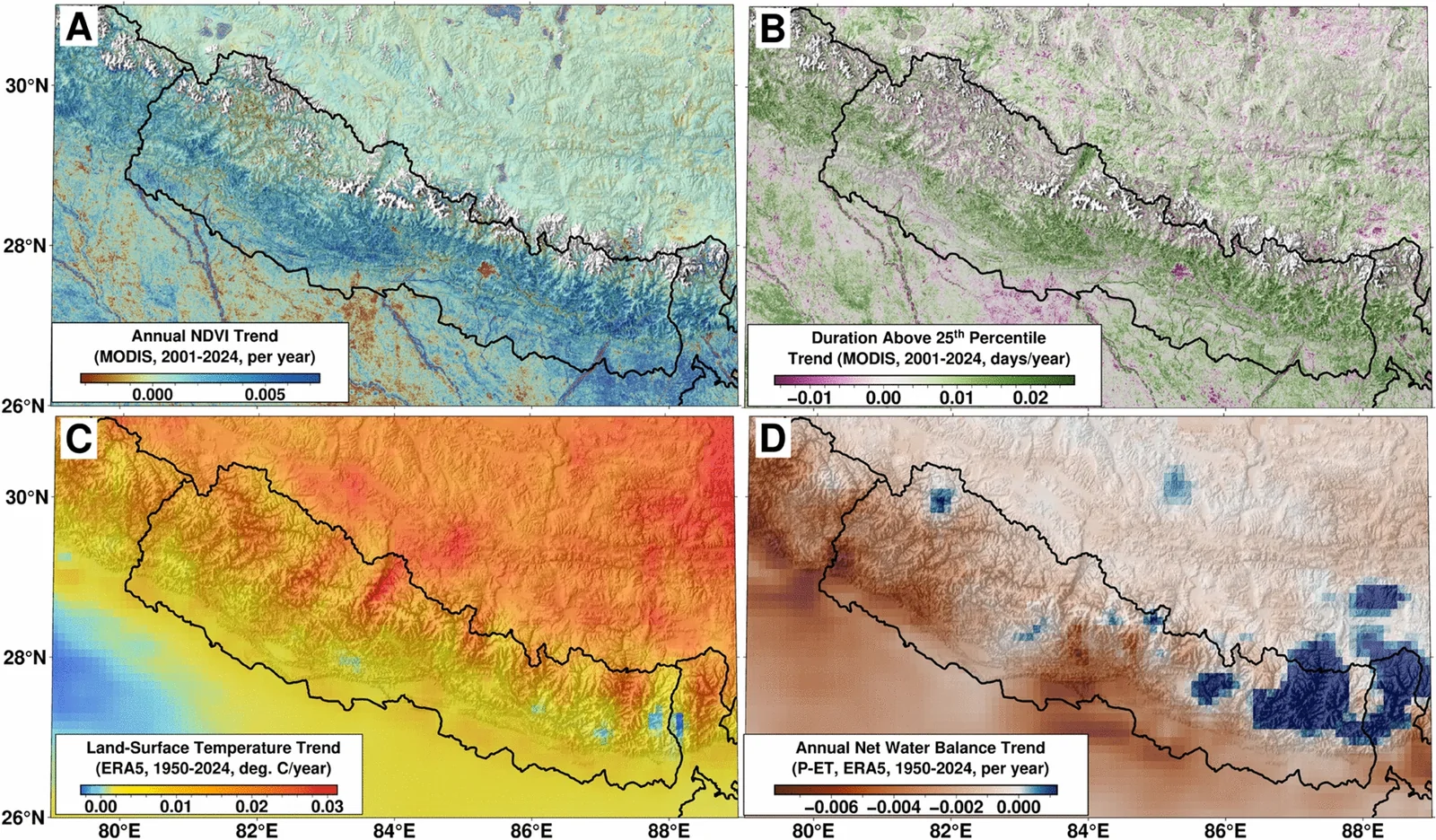
Long-term changes in environmental water use. (**A**) Long-term NDVI trend (MODIS, 2001–2024^19^). (**B**) Trend in duration above 25th percentile NDVI (MODIS, 2001–2024^19^). We observe positive trends in both parameters, suggesting an increase in chlorophyll content and productive season length. (**C**) Land-surface temperature trend (ERA5, 1950-2024^18^) is positive throughout the study area with higher trends at higher elevations^47^ (Supplemental Figure S16). (**D**) Regional water balance trends (total precipitation - evaporation, ERA5, 1950–2024^18^) are negative with a few positive exceptions near glaciated areas. Maps generated with pygmt (0.17.0)^36^ using the Copernicus DEM as background^37^.

Ongoing environmental changes linked to precipitation shifts, temperature increases, and vegetation changes are another potential driver (Fig. 5). We find broadly increasing vegetation productivity throughout the region, as well as an increased productive vegetation duration. These two factors together could lead to more plant-water uptake, decreasing the availability of upstream water for groundwater and downstream discharge. Warming has been well-documented in the region^1^ (Fig. 5c); increased temperatures have decreased snow persistence, decreased soil moisture, and encouraged the upward migration of vegetation into previously bare regions. Each of these factors could play a role in changing discharge characteristics, with varied influence on rainfall-discharge and snowmelt-discharge correlations. At the broad scale, increased soil moisture deficits due to rising temperatures are likely to reduce precipitation conversion into downstream discharge, regardless of whether it is sourced as rainfall or snowmelt. The lack of increasing early-season discharge (Fig. 1) or shifts in the seasonal discharge cycle (Fig. 2) imply that snowmelt changes are not strongly propagating into discharge in our study area.

Changes in NDVI and growing season length are positive across all elevation bins (Supplemental Figure S16), though the strongest positive vegetation trends are at low elevations. We further find that temperature trends are positive across all elevations, with the highest trends at high elevations. Finally, water deficits are also increasing across all elevation bins, with the largest increases at low elevations (Supplemental Figure S16). We thus conclude that changes in water use and stress are not confined to only some parts of each watershed, but are rather a broad-based phenomenon that is impacting water usage throughout our study region. As these environmental changes are present over entire watersheds, their expected impact is substantial; we propose that these environmental changes are a primary driver of diminishing discharge throughout our study area.

One additional factor that has been widely discussed in High Mountain Asia is changes in glacier contribution to discharge^3,15,48–50^, especially in the Indus basin. Previous investigations have suggested that while discharge observations in large rivers may be biased — especially in the Indus River^15^ — there is a general trend towards higher discharge in the northwestern Himalaya^48^, alongside decreasing groundwater storage^51^. Increasing discharge has been linked to increasing snow and glacier melt, especially in the early summer season^48^. We do not observe increased discharge in our study region (Fig. 1), even in the higher-elevation, sparsely vegetated, and glacier-covered catchments which are similar to those studied in the northwestern Himalaya. Despite increased spring snowmelt in many catchments in our study area, we do not see increased spring discharge (Fig. 1). We thus posit that large-scale environmental drivers (e.g., vegetation cover, soil-water deficits, increasing temperatures) are a more important control on precipitation-discharge correlations than changes in glacier and snow-water resources in the Central Himalaya.

### Correlation and transit time links

**Fig. 6:**
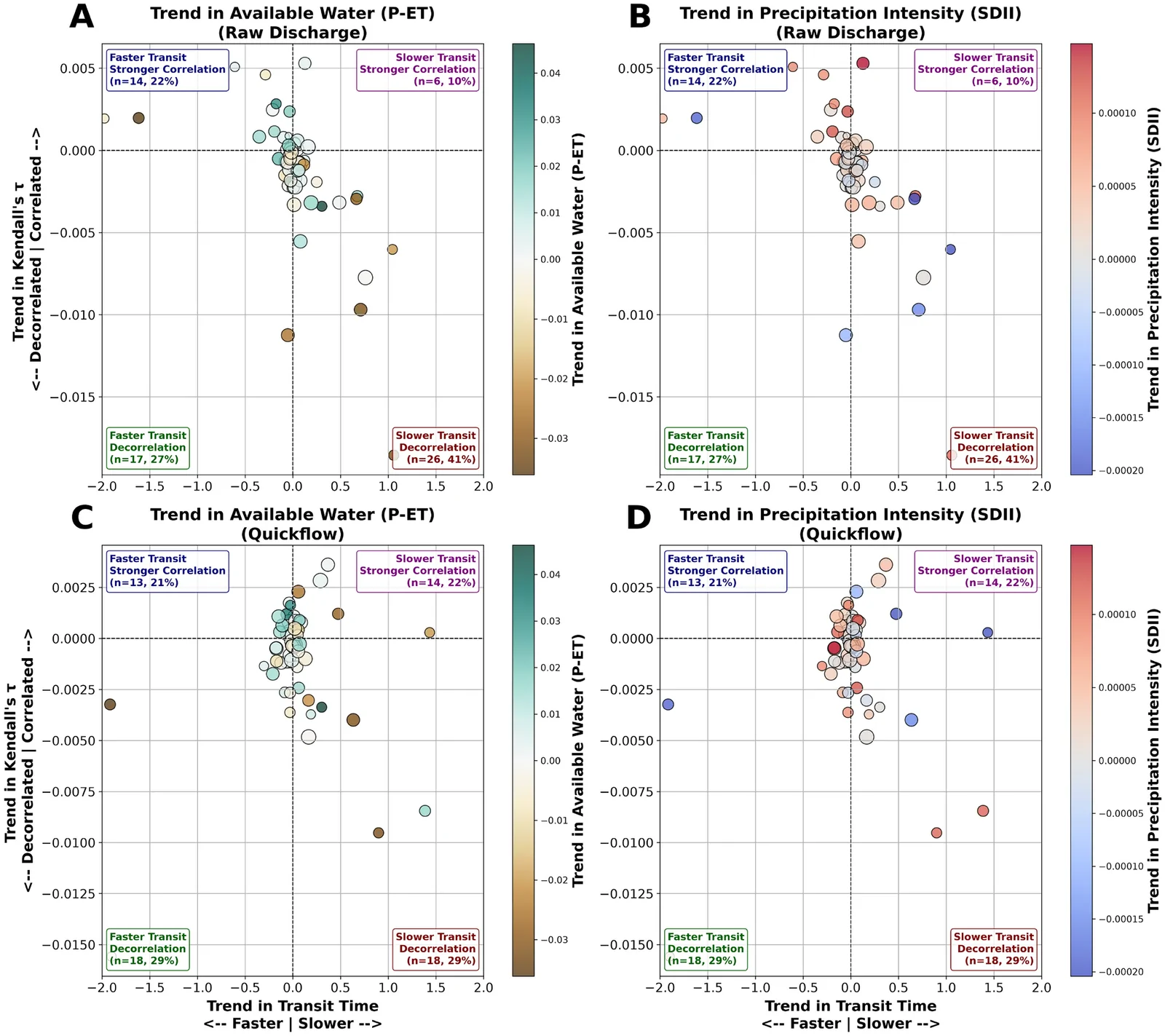
Rainfall-discharge trend classification. Scatter plots of rainfall-discharge correlation trend direction compared to two potential drivers: (**A,C**) available water and (**B,D**) precipitation intensity. Only points with significant Kendall’s *τ * and transit time trends are shown. The number of points in each quadrant is shown in the corners of each plot. Dots sized by catchment drainage area. (**A,B**) show raw discharge relationships, (**C,D**) show quickflow-discharge relationships.

If we consider both changes in precipitation-discharge correlation and transit time together, we can better classify the changes in basin-scale water transport. For example, catchments that have decreased transit times and increased correlation can be considered to be ‘flashier’ and more rapidly convert water into discharge. Conversely, slower transit times and weaker correlation would indicate that water is taking longer to move through the catchment, and acting more like a sink. The majority of catchments fall into these two bins for rainfall-discharge relationships (Fig. 6a,b).

While there remains noise in the data, there is a tendency for ‘flashier’ catchments to have positive trends in available water and precipitation intensity (Supplemental Table S3). We hypothesize that these catchments are converting any increased rainfall more rapidly into discharge. Conversely, slowing-transport catchments are acting more like sinks, and correlate with higher water deficits and less intense precipitation (Fig. 6a,b). Both the ‘flashier’ catchments and ‘sink’ catchments are well-distributed throughout Nepal and cannot be succinctly grouped by land cover, elevation, or climate setting.

Only six catchments show an increased correlation and increased transit time; one potential mechanism would be longer flow paths (e.g., via hydropower reservoirs) alongside a linear response to direct precipitation. These outlier stations are all low-elevation and small catchments which are located in vegetated areas with substantial rainfall. Comparatively more catchments show a signal of decreased correlation and decreased transit times, which could be a sign of more chaotic or threshold-based flow due to, e.g., human landscape modifications; these stations are distributed across Nepal, though tend to be some of the larger catchments examined. We note that these catchments — excepting one short time series — all have time series longer than 20 years, indicating the outliers are not simply due to short data records. The majority of catchments in the lower left and upper right quadrants (Fig. 6), however, have very low trends, making their interpretation more difficult.

When we compare the correlation-transit time data to potential explanatory variables (Fig. 6a,b), we find that slowing and decorrelating catchments tend to have higher water deficits (mean: -0.001 m/year) and decreasing precipitation intensities (mean: $-2.1\text {e-}06$ mm/day/year). Both factors have the potential to impact discharge – less intense but steady precipitation would likely take longer flow paths (e.g., through soil absorption and groundwater) and hence lead to longer transit times, especially if soil-moisture deficits are increasing due to plant-water uptake and increasing air temperatures (Fig. 5). Conversely, if rainfall occurs as more intense and shorter storms, it is likely to lead to more overland flow and hence a more direct rainfall-discharge relationship. We lack, however, enough time-resolved data on changes in water budgets over the entire region to definitively link changes in coupled correlation-transit time to a set of mechanisms; further work would be needed to confirm the direct and coupled impacts of precipitation and water deficit changes on discharge throughout the region.

If we repeat this analysis using quickflow (discharge with annual baseflow removed), the dominance of ‘flashier’ and sink-like catchments decreases (raw discharge: 63% of catchments; quickflow: 50% of catchments) (Fig. 6c,d; Supplemental Table S4), and the other pairings (upper-right and lower-left, Fig. 6) become more pronounced. This suggests that the diagonal clustering in Fig. 6 is to some degree controlled by the low-pass filtering and storage-buffering influence of slow subsurface flow. The upper-right quadrant (slower transit times and stronger correlation) jumps from 6 to 14 stations, and the lower-left quadrant (faster transit times and weaker correlation) increases slightly from 17 to 18 stations while falling further away from the vertical zero line (Fig. 6). However, the conclusions we draw from this analysis largely support our findings based on raw discharge.

Stations in the upper-right quadrant (slower transit times, stronger correlation) shift from positive to negative available water and SDII trends when using quickflow. This indicates that when the influence of baseflow is removed, storm routing is more strongly influenced by increased water storage capacity in dry soils, which can delay quickflow routing (longer transit times) while still keeping strong event-based coupling (i.e., storm runoff limited to high-magnitude events that saturate dry soils). The lower-left quadrant (faster transit times and weaker correlation) shows a much more strongly negative transit time trend (quickflow: -0.29; raw discharge: -0.04 days/year) with similar decoupling (quickflow: -0.002; raw discharge: -0.002). These stations are linked to heavier precipitation, which could indicate that quickflow is a threshold-driven process in these catchments where intense storms are quickly routed into rivers (decreasing transit times); such a process could reduce overall correlation if the quickflow routing was strongly non-linear (i.e., only after a certain size storm is there a strong discharge response).

The ‘flashier’ catchments (faster transit times, stronger correlation) exhibit an even stronger trend towards more available water (quickflow: 0.01; raw discharge: 0.005 m/year), alongside positive SDII trends ($3.2\text {e-}05$ mm/day/year). Conversely, the ‘sink’ catchments (slower transit times, lower correlation) are no longer strongly correlated with drying (raw discharge: -0.001; quickflow: 0.002 m/year) and decreased SDII (raw discharge: $-2.1\text {e-}06$; quickflow: $2\text {e-}05$ mm/day/year), but rather neutral water availability and SDII. This indicates that baseflow reductions are more important in driving the ‘sink’ behavior than rapid quickflow storm response; this interpretation agrees well with our overall interpretation that greening and warming trends are increasing soil-water uptake and decreasing the amount of available water for direct discharge.

Changes in the precipitation-discharge relationship will likely be felt downstream, and these changes will not always have a negative impact. Increased transit times and a smoothing of the rainfall-discharge relationship imply diminished peak flow intensities as more water is intercepted by vegetation and transiently stored in soil and groundwater reservoirs. This increased buffering could help offset declines in snowmelt as precipitation shifts towards rainfall in some catchments, as well as reduce the impact of intensifying precipitation in the region^52^. In already-dry catchments, however, increased evaporative water demand and the general decrease in discharge (Fig. 1) will influence ecosystems and communities that depend on rivers. Further work is needed to better constrain how changing land cover will shape discharge in the coming decades.

## Conclusions

Throughout the Central Himalaya, the relationship between discharge and upstream water (snowmelt, precipitation) is shifting; we find an increase in upstream and decrease in downstream water flux. Here, we examine long-term in-situ discharge data alongside watershed-averaged upstream climate and environmental information to untangle the drivers of divergent precipitation-discharge trends. We find a widespread shift towards reduced precipitation-discharge correlation and longer transit times between upstream inputs and downstream discharge. These trends are suggestive of wide-scale environmental changes that are impacting the water budget of Central Himalayan catchments; we posit that warming, increased vegetation productivity, and longer growing seasons are primary drivers of increased soil-water uptake, which translates into diminishing discharge despite increased precipitation. These changes have made many of the catchments studied here act more like sinks, where more water is used before it reaches the river and water takes longer paths through the catchment.

Understanding changes in the relationship between climate, precipitation, and discharge will be key to regional water planning in the coming years. This is of particular importance given the growing role of hydropower in national energy systems in the region. While the implied changes in watershed hydrology found here are likely disruptive to natural ecosystems, in some cases, they may be rather beneficial to human users. In particular, rainfall-discharge decoupling, alongside only slight changes in rainfall, implies a smoothing of the hydrograph, potentially minimizing the potential for flood events and increasing the ability of local communities to draw on rivers, despite the decreases in average discharge.

## Supplementary Information


Supplementary Information.


## Data Availability

ERA5 data can be found here: https://doi.org/10.24381/cds.e2161bac. MODIS NDVI Data (MOD13Q1) can be found here: https://doi.org/10.5067/MODIS/MOD13Q1.061. Both data sets can also be accessed via Google Earth Engine. Human Footprint Index data can be found here: https://doi.org/10.6084/m9.figshare.16571064. Nepali hydropower projects are listed here: https://doed.gov.np/pages/powerplantsmorethan1/; we only include dams with a commissioning date and a well-located spatial coordinate. Microsoft Building Footprints data is found here: https://github.com/microsoft/GlobalMLBuildingFootprints/. OpenStreetMap data is available here: https://download.geofabrik.de/asia-latest.osm.pbf, we collected our data in April 2025 using the ‘latest’ data at that point. Landslide data is available here: https://bipadportal.gov.np/incidents/ by exporting all ‘landslide’ events as a CSV file (Accessed April 2025). Discharge data was obtained from the Department of Hydrology and Meteorology, Nepal, and can be accessed from them by request: https://dhm.gov.np/pages/data.

## Code availability

Python code used to pre-process and export the data via Google Earth Engine is available on Zenodo: https://doi.org/10.5281/zenodo.21493188. Code to analyze discharge and environmental data in concert is also available on Zenodo.
