# Supplementary material for: Discharge deficits in the Central Himalaya linked to greening and warming
Source: Sci Rep. 2026 Sep 1;16:27373. doi: 10.1038/s41598-026-68997-3 (PMC13530239; doi:10.1038/s41598-026-68997-3)
Supplement: Supplementary file 1 [file 41598_2026_68997_MOESM1_ESM.pdf]

Supplement to: Discharge Deficits in the Central Himalaya Linked to Greening and Warming

Taylor Smith[1] and Bodo Bookhagen[1]
[1]Institute of Geosciences, Universität Potsdam, Germany

Corresponding author:
Taylor Smith
Email: tasmith@uni-potsdam.de

List of Figures

S1 **Data availability**. Each available station is listed on the y-axis, with available data per day shown in black (long records) or gray (short records). Only stations with more than 10 years of data are used in our analysis. 3

S2 **5th Percentile Discharge and Precipitation Trends**. Changes in (A) Discharge, (B) Total Precipitation, (C) Spring Discharge, and (D) Spring Snowmelt. Annual aggregation to 5th percentile before trend calculation. 6

S3 **95th Percentile Discharge and Precipitation Trends**. Changes in (A) Discharge, (B) Total Precipitation, (C) Spring Discharge, and (D) Spring Snowmelt. Annual aggregation to 95th percentile before trend calculation. 6

S4 **Example Discharge Distribution**. Discharge z-scores over a short period, showing a subset of stations ranked from high to low elevation. Seasonal discharge distributions throughout the year follow a similar pattern across Nepal. 7

S5 **Example Time Series Processing**. Left panels: ERA5 data. Right panels: GLDAS data. (A) Discharge data with different correction schemes. (B) Rainfall and Snowmelt time series. (C) Rolling Kendall's τ statistics, with linear fits. (D) Rolling transit time statistics, with linear fits. 8

S6 **Changes in Accumulated Discharge Timing**. Day of year trends in the timing of discharge accumulation for the thresholds of (A) 25th percentile and (B) 50th percentile. Discharge tends to be accumulating later in the year across much of the study area. 8

S7 **Seasonal Correlation.** (A) Seasonal (90-day rolling) Kendall's τ statistics for rainfall (blue) and snowmelt (orange) correlated with discharge. Averaged by day-of-year and over all watersheds and all station-years; the number of station-years is summed in the legend. (B) Rainfall and (C) snowmelt Kendall's τ split by decade, showing generally similar seasonal correlation structures through time. Discharge data processed to remove annual baseflow. 9

S8 **Inter-Annual correlation.** Kendall's τ averages (three-year window) for (A) rainfall and (B) snowmelt. 2000 m elevation contour shown as purple line. Dots sized by catchment drainage area. Average transit times (three-year window) for (C) rainfall and (D) snowmelt. Discharge data processed to remove annual baseflow. 9

S9 **Inter-Annual correlation.** Kendall's τ averages (three-year window) for (A) rainfall and (B) snowmelt. 2000 m elevation contour shown as purple line. Dots sized by catchment drainage area. Average transit times (three-year window) for (C) rainfall and (D) snowmelt. Discharge data processed to correct rating-curve shifts using BEAST. 10

S10 **Inter-Annual correlation.** Kendall's τ averages (three-year window) for (A) rainfall and (B) snowmelt. 2000 m elevation contour shown as purple line. Dots sized by catchment drainage area. Average transit times (three-year window) for (C) rainfall and (D) snowmelt. Discharge data compared to GLDAS instead of ERA5 data. 10

S11 **Precipitation-Discharge Correlation Changes**. Kendall's τ trend between rainfall and discharge (A) and snowmelt and discharge (B). 2000 m elevation contour shown as purple line. Dots sized by catchment drainage area. Trend in transit time for (C) rainfall and (D) snowmelt. Discharge data processed to correct rating-curve shifts using BEAST. 11

S12 **Precipitation-Discharge Correlation Changes**. Kendall's τ trend between rainfall and discharge (A) and snowmelt and discharge (B). 2000 m elevation contour shown as purple line. Dots sized by catchment drainage area. Trend in transit time for (C) rainfall and (D) snowmelt. Discharge data processed to remove annual baseflow. 11

S13 **Precipitation-Discharge Correlation Changes**. Kendall's τ trend between rainfall and discharge. 2000 m elevation contour shown as purple line. Dots sized by catchment drainage area. (A) Raw data, (B) BEAST correction, (C) CFA correction. 12

S14 **Precipitation-Discharge Correlation Changes**. Kendall's τ trend between rainfall and discharge. 2000 m elevation contour shown as purple line. Dots sized by catchment drainage area. (A) ERA5 data, (B) GLDAS data, (C) GPM data, (D) CHIRPS data. 12

S15 **Impact of Human Infrastructure**. Comparison of Rainfall-Discharge Kendall's τ trends to upstream infrastructure (A: roads, B: buildings, C: Human Footprint Index) and (D) landslide frequency. The relationships between landscape modification and correlation trends are generally weak. 13

S16 **Role of Hypsometry in Environmental Trends**. Comparison of (A) NDVI, (B) LST, and (C) Water Balance trends to hypsometry (250 m elevation bins). Each binned average line normalized to the maximum to show relative differences. Low elevations tend to have lower LST trends but stronger NDVI and water balance changes. 14

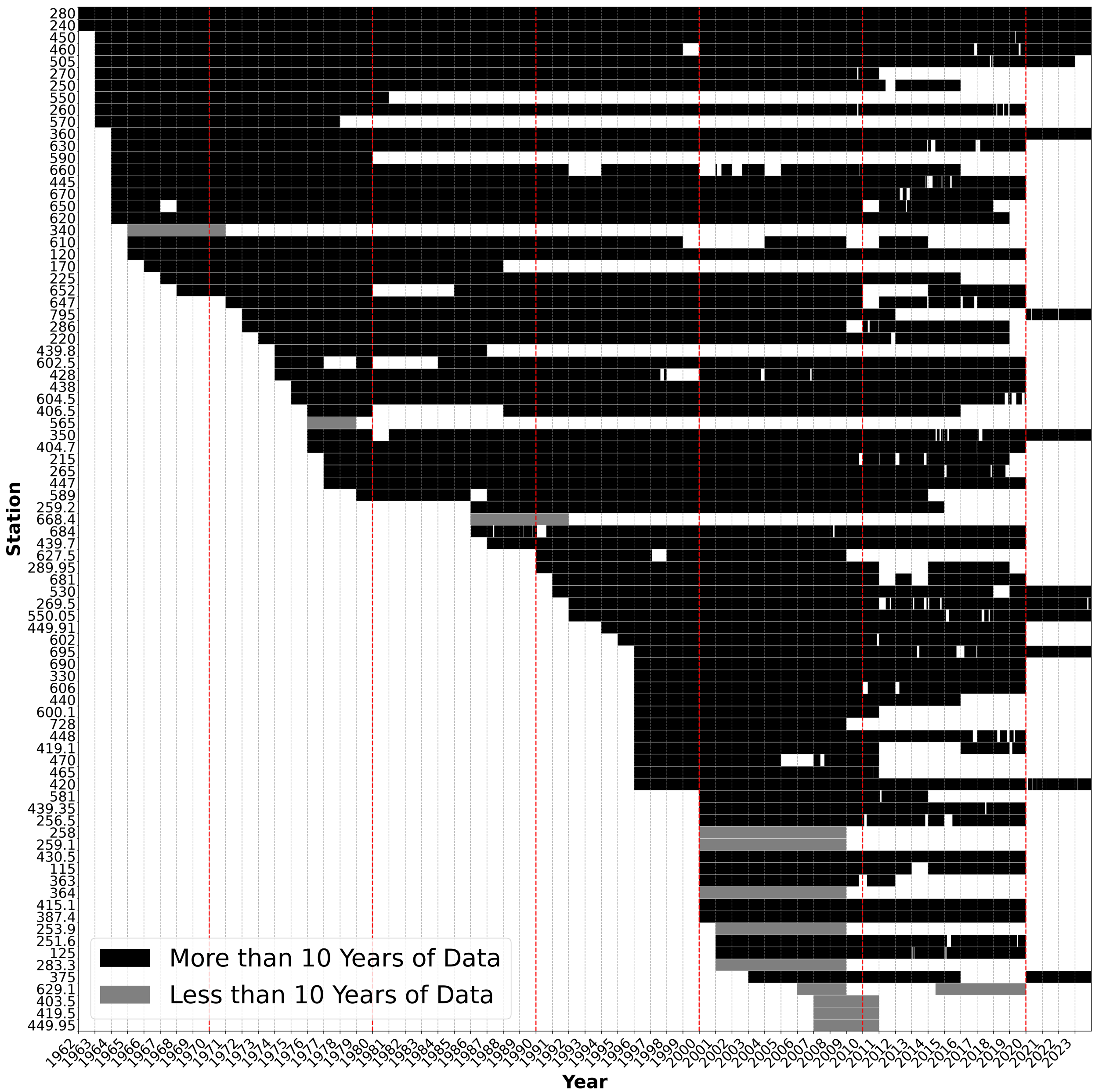

Figure S1: **Data availability**. Each available station is listed on the y-axis, with available data per day shown in black (long records) or gray (short records). Only stations with more than 10 years of data are used in our analysis.

Table S1: Discharge record information.

Station	Lat.	Lon.	River	Rec. Len. (yr)	Missing (%)	Max Gap (d)
115	29.702	80.607	Naugra gad	20	11.4	365
120	29.672	80.558	Chamelia	55	4.7	335
215	29.159	81.600	Humla Karnali	42	1.9	91
220	29.200	81.917	Tila Nala	46	0.5	89
225	29.200	81.917	Sinja Khola	49	0.7	120
240	28.953	81.442	Karnali River	62	0.4	53
250	28.961	81.119	Karnali River	53	1.2	215
259.2	29.300	80.775	Seti River	29	0.1	15
260	28.978	81.144	Seti River	57	0.9	31
265	28.713	82.292	Thulo Bheri	42	2.6	153
269.5	28.517	81.667	Bheri	32	3.8	151
270	28.756	81.350	Bheri River	48	1.2	184
280	28.644	81.292	Karnali	62	0.8	169
286	28.299	82.025	Sarada Khola	47	3.2	365
289.95	28.351	81.733	Babai	29	11.3	1096
330	28.072	82.800	Mari Khola	24	0.1	2
350	27.900	82.850	Rapti River	48	3.1	366
360	27.947	82.225	Rapti River	60	0	0
363	28.156	81.754	Jhanjhari	12	15.8	186
375	28.001	82.116	Rapti	21	19	1461
387.4	27.796	83.536	Dumre Khola	20	0.4	28
406.5	28.254	83.724	Modi Khola	40	22.1	2922
415.1	27.974	83.574	Andhi Khola	20	3.2	73
419.1	27.885	83.795	Kali Gandaki	24	21.6	1826
420	27.750	84.347	Kali Gandaki	28	1.4	29
430.5	27.933	84.250	Seti Gandaki	20	0	0
438	28.100	84.233	Madi River	45	1.7	200
439.35	28.204	84.403	Marsyangdi River	20	1	31
439.7	27.950	84.430	Marsyangdi	33	4.7	146
439.8	27.926	84.495	Marsyangdi	13	0	0
440	28.061	84.490	Chepe Khola	20	1.7	13
445	28.044	84.816	Burhi Gandaki	56	1.7	152
447	27.969	85.183	Trishuli	43	0.1	3
448	27.860	85.138	Tadi Khola	24	3.1	92
449.91	27.836	84.553	Trishuli	26	1.5	127
450	27.708	84.431	Narayani	61	0.8	122
460	27.447	84.974	Rapti River	61	5	365
465	27.550	84.817	Manahari Khola	15	4.9	34
470	27.587	84.735	Lothar Khola	15	22.3	883
505	27.775	85.428	Bagmati River	60	6.4	131
550	27.661	85.297	NaN	18	0	1
550.05	27.629	85.295	Bagmati	32	1.5	70
570	27.586	85.158	Kulekhani Khola	15	0.8	12
581	27.362	85.469	Bagmati River	14	0.5	27
589	27.152	85.492	Bagmati River	35	5.8	499
590	27.106	85.475	Bagmati River	16	4.1	87
600.1	27.600	87.339	Arun River	15	0	2
602	27.310	87.221	Sabhaya Khola	25	1.3	45
602.5	27.296	87.225	Hinwa Khola	46	16.2	1475
604.5	27.333	87.192	NaN	45	2.8	142
606	26.928	87.158	NaN	24	2.5	115
610	27.788	85.899	Bhote Kosi	49	16.7	2023
620	27.806	85.769	Balephi Khola	55	1	94
627.5	28.042	85.535	Melamchi Khola	19	5.6	323
630	27.558	85.753	Sun Kosi	56	2.9	209
647	27.635	86.087	Tamakosi	49	7.3	471
650	27.575	86.197	Khimti Khola	54	7	411
652	27.336	86.000	Sunkosi	52	23.3	1827
660	27.336	86.219	Likhu Khola	52	14.1	731
670	27.271	86.667	Dudh Kosi	56	2.5	245
681	26.921	87.146	Sun Kosi	29	7	365
684	27.158	87.713	Tamur	34	3	210
690	26.931	87.329	Tamur River	24	1.7	124
728	26.879	87.929	Mai Khola	13	1.9	59
795	26.687	87.879	Kankai Mai River	52	15.5	2922

Table S2: Catchment characteristics.

Station	Lat.	Lon.	River	Area (km^2)	Min Elev. (m)	Mean Elev. (m)	Max Elev. (m)	Snowfall Fraction (%)
115	29.702	80.607	Naugra gad	205.4	811	2290.9	3952	7.6
120	29.672	80.558	Chamelia	1158.1	683	3012	7076	19
215	29.159	81.600	Humla Karnali	16954.7	745	4293.4	7645	62
220	29.200	81.917	Tila Nala	1750.6	2038	3591.1	6493	24.6
225	29.200	81.917	Sinja Khola	1750.6	2038	3591.1	6493	24.6
240	28.953	81.442	Karnali River	20845.1	627	4073.8	7645	46.8
250	28.961	81.119	Karnali River	22646.7	330	3862.8	7645	39.4
259.2	29.300	80.775	Seti River	4305.4	622	3031.2	7015	17.2
260	28.978	81.144	Seti River	7357.2	341	2520.5	7015	10.2
265	28.713	82.292	Thulo Bheri	11.6	1064	2019.1	2907	0.9
269.5	28.517	81.667	Bheri	12660.9	416	3271.9	7735	16.3
270	28.756	81.350	Bheri River	10.4	285	648.4	1073	0
280	28.644	81.292	Karnali	45436.9	191	3304	7735	21.7
286	28.299	82.025	Sarada Khola	812.3	627	1439.9	2815	0
289.95	28.351	81.733	Babai	2596.2	311	1025.3	2815	0
330	28.072	82.800	Mari Khola	1958.3	529	1813.2	3651	0.8
350	27.900	82.850	Rapti River	3589.5	332	1589.9	3651	0.5
360	27.947	82.225	Rapti River	5141.5	211	1261.2	3651	0.4
363	28.156	81.754	Jhanjhari	73.5	155	445.4	1124	0
375	28.001	82.116	Rapti	5285	204	1237.7	3651	0.4
387.4	27.796	83.536	Dumre Khola	75.9	607	1174.3	1893	0
406.5	28.254	83.724	Modi Khola	559.1	816	3469.5	7958	9.5
415.1	27.974	83.574	Andhi Khola	7589	500	3611.8	8134	16.4
419.1	27.885	83.795	Kali Gandaki	10627.7	341	3032.5	8134	10.3
420	27.750	84.347	Kali Gandaki	11822.6	198	2804.3	8134	8.8
430.5	27.933	84.250	Seti Gandaki	1470.6	311	1621.6	7532	3.1
438	28.100	84.233	Madi River	852.3	386	2331.2	7883	5.6
439.35	28.204	84.403	Marsyangdi River	3003.6	631	4377.1	8040	39.5
439.7	27.950	84.430	Marsyangdi	4058.5	350	3707.4	8040	24.1
439.8	27.926	84.495	Marsyangdi	4115.3	336	3665.5	8040	23.8
440	28.061	84.490	Chepe Khola	309.8	443	1819.8	4949	1.7
445	28.044	84.816	Burhi Gandaki	3876.6	481	4265.4	8041	47.6
447	27.969	85.183	Trishuli	4646.1	597	4346.6	7351	35.9
448	27.860	85.138	Tadi Khola	653.1	473	1648.3	5073	1
449.91	27.836	84.553	Trishuli	16723.7	219	3449.5	8041	21.7
450	27.708	84.431	Narayani	31742	179	3019	8134	14
460	27.447	84.974	Rapti River	444.4	394	951.4	2502	0
465	27.550	84.817	Manahari Khola	427.7	303	1151.5	2589	0
470	27.587	84.735	Lothar Khola	169.1	270	930.8	2080	0
505	27.775	85.428	Bagmati River	15.6	1613	2100.9	2723	0.2
550	27.661	85.297	NaN	587.8	1268	1541.1	2726	0.1
550.05	27.629	85.295	Bagmati	608.4	1246	1538.8	2726	0.1
570	27.586	85.158	Kulekhani Khola	125	1449	1942.2	2588	0
581	27.362	85.469	Bagmati River	1704.4	265	1468.9	2930	0
589	27.152	85.492	Bagmati River	2819.2	129	1090.5	2930	0
590	27.106	85.475	Bagmati River	2835.4	118	1085.7	2930	0
600.1	27.600	87.339	Arun River	29983.7	868	4889.6	8737	61.4
602	27.310	87.221	Sabhaya Khola	148.3	315	1532	3701	0.7
602.5	27.296	87.225	Hinwa Khola	149.8	305	1521	3701	0.7
604.5	27.333	87.192	NaN	31511.3	288	4773.7	8737	49.3
606	26.928	87.158	NaN	33821.8	123	4542.5	8737	37.1
610	27.788	85.899	Bhote Kosi	2377.5	845	4569.6	7918	52.7
620	27.806	85.769	Balephi Khola	622.8	787	3348.3	6903	15
627.5	28.042	85.535	Melamchi Khola	74.2	2319	4314.3	5807	12.4
630	27.558	85.753	Sun Kosi	4864.4	586	3430.5	7918	19.7
647	27.635	86.087	Tamakosi	2948.5	844	4245.8	7299	29.4
650	27.575	86.197	Khimti Khola	326.4	1544	2769.8	5403	3.1
652	27.336	86.000	Sunkosi	10215.3	452	3259.2	7918	15.9
660	27.336	86.219	Likhu Khola	858.5	546	2829.3	6842	4.1
670	27.271	86.667	Dudh Kosi	3740.1	446	3744.6	8727	13.5
681	26.921	87.146	Sun Kosi	18260.6	121	2952.4	8727	11.1
684	27.158	87.713	Tamur	4046.8	419	3522.6	8555	13.9
690	26.931	87.329	Tamur River	5940.8	251	2870.1	8555	9
728	26.879	87.929	Mai Khola	382.6	440	1729.1	3619	0
795	26.687	87.879	Kankai Mai River	1177.8	115	1242.3	3619	0

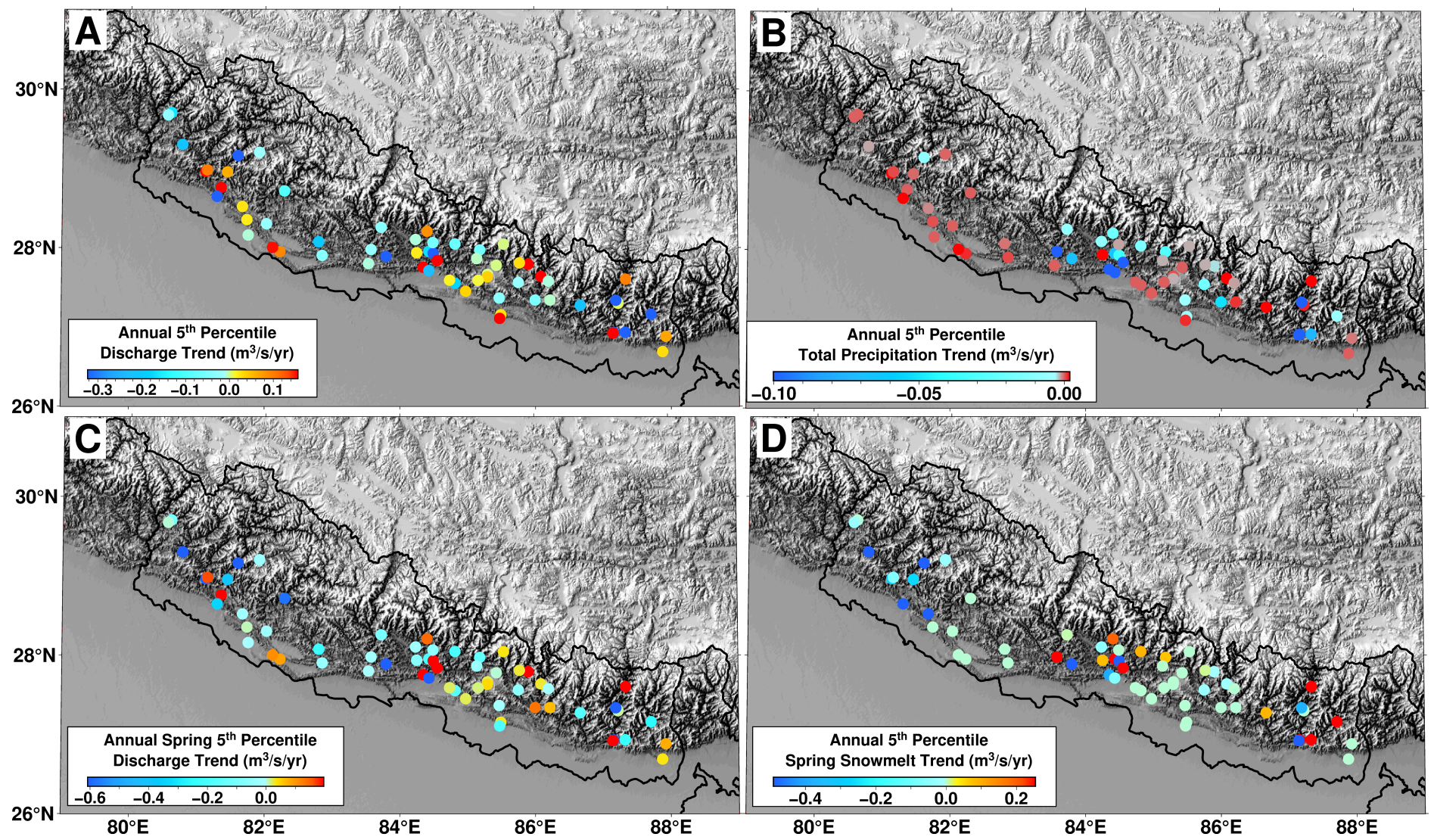

Figure S2: **5th Percentile Discharge and Precipitation Trends**. Changes in (A) Discharge, (B) Total Precipitation, (C) Spring Discharge, and (D) Spring Snowmelt. Annual aggregation to 5th percentile before trend calculation.

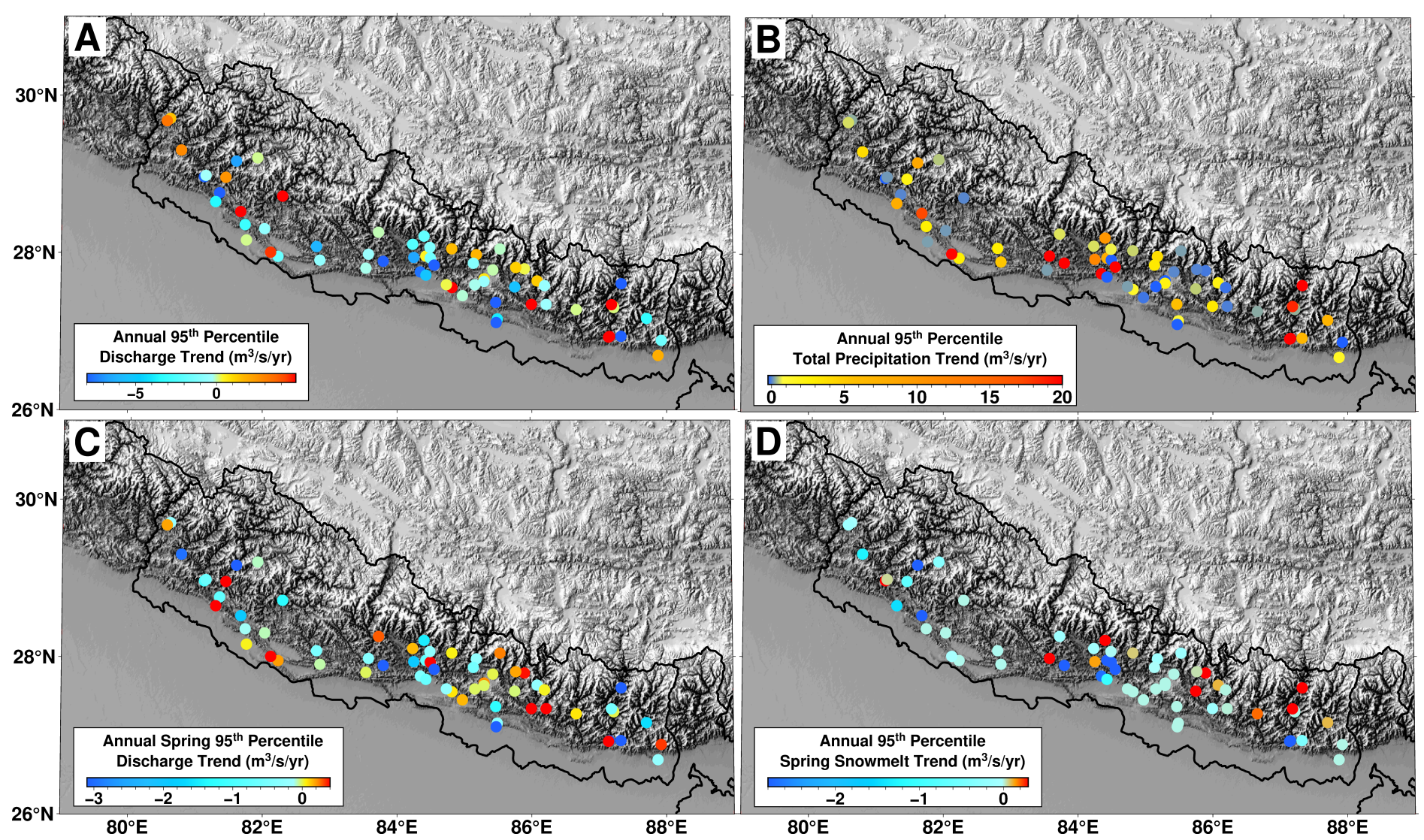

Figure S3: **95th Percentile Discharge and Precipitation Trends**. Changes in (A) Discharge, (B) Total Precipitation, (C) Spring Discharge, and (D) Spring Snowmelt. Annual aggregation to 95th percentile before trend calculation.

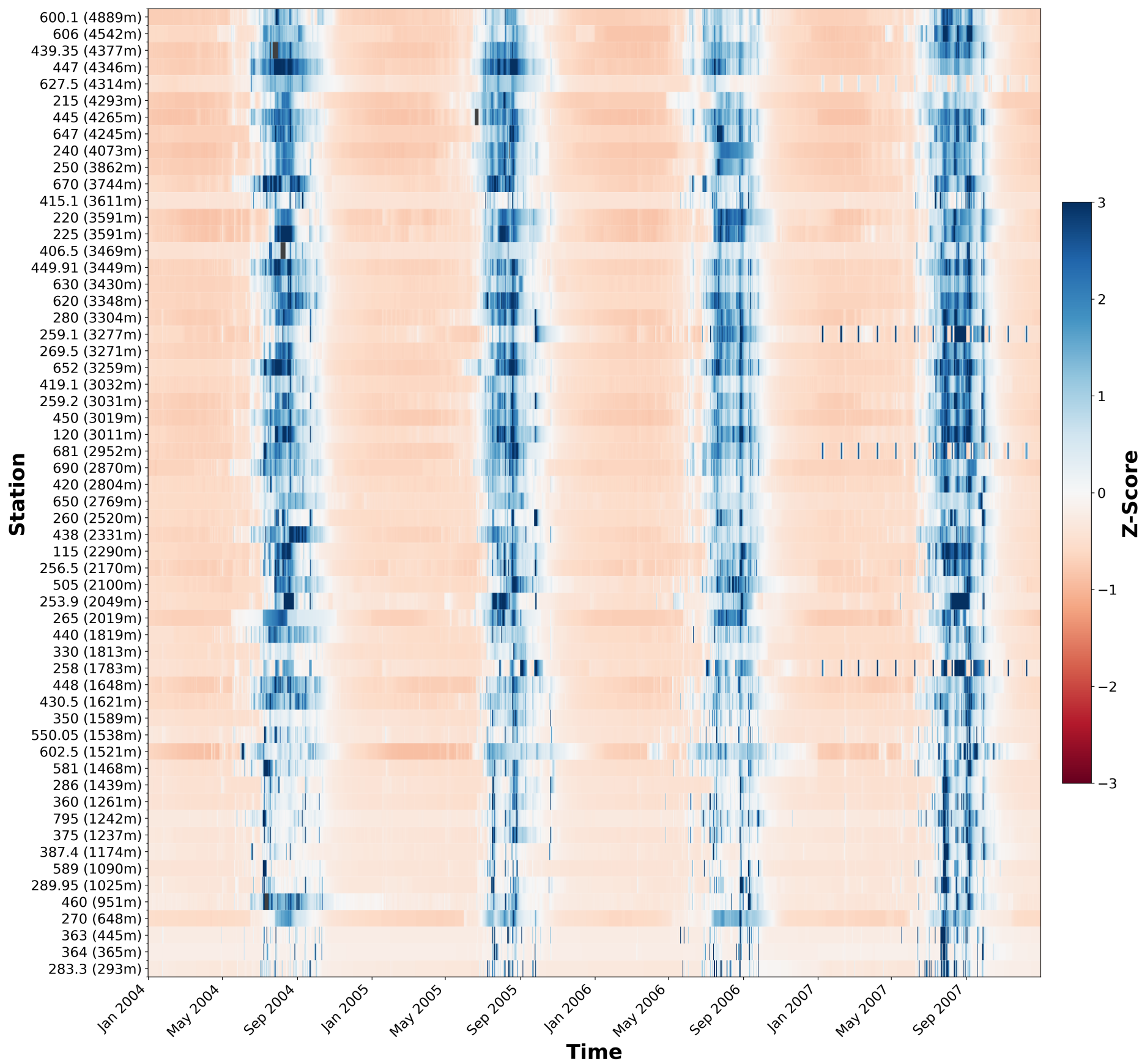

Figure S4: **Example Discharge Distribution**. Discharge z-scores over a short period, showing a subset of stations ranked from high to low elevation. Seasonal discharge distributions throughout the year follow a similar pattern across Nepal.

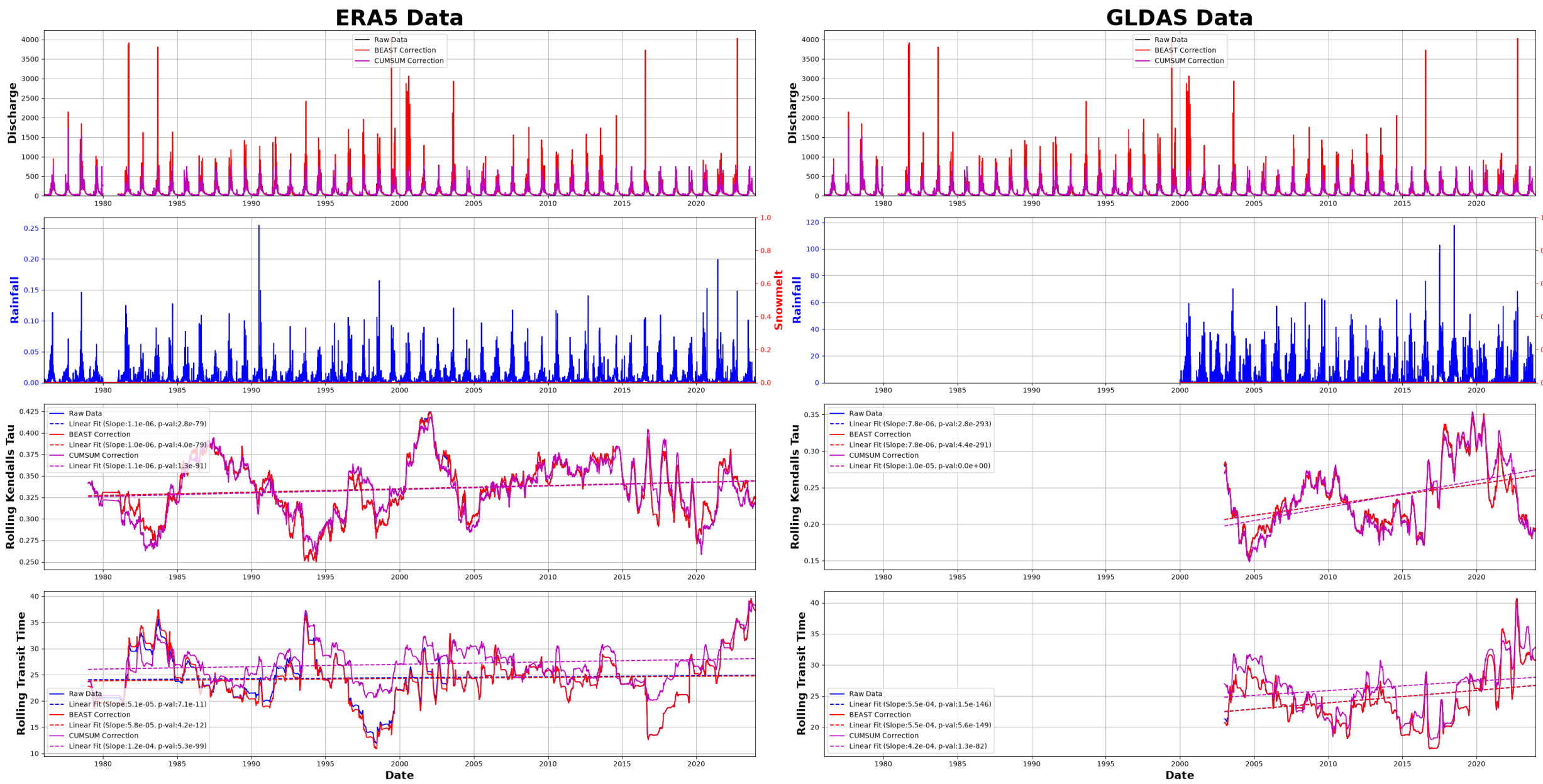

Figure S5: **Example Time Series Processing**. Left panels: ERA5 data. Right panels: GLDAS data. (A) Discharge data with different correction schemes. (B) Rainfall and Snowmelt time series. (C) Rolling Kendall's τ statistics, with linear fits. (D) Rolling transit time statistics, with linear fits.

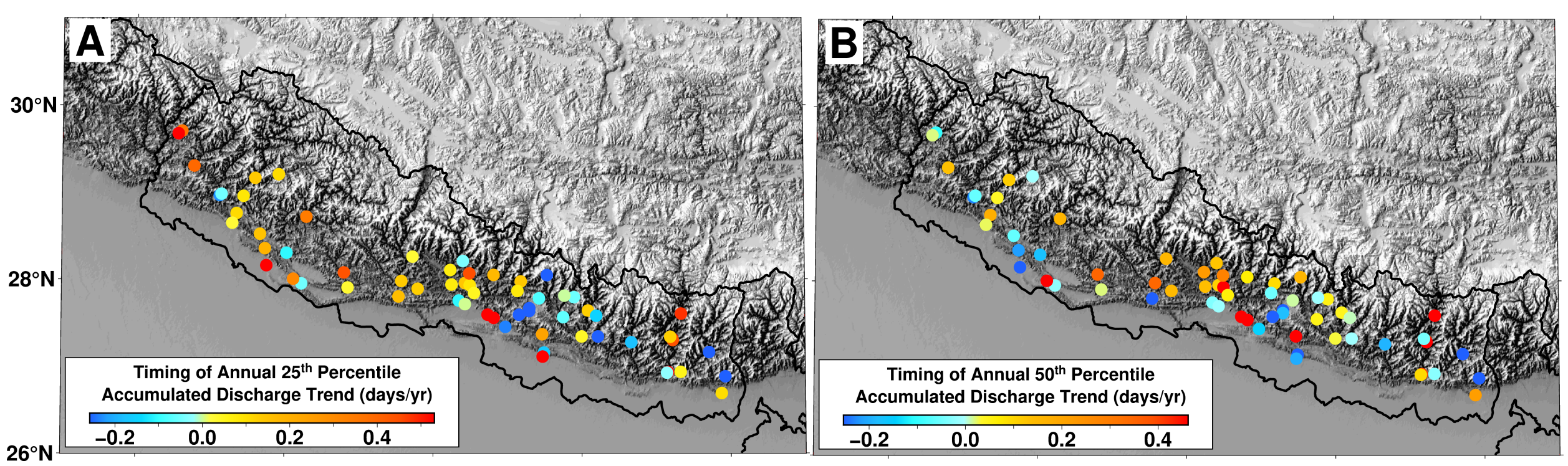

Figure S6: **Changes in Accumulated Discharge Timing**. Day of year trends in the timing of discharge accumulation for the thresholds of (A) 25th percentile and (B) 50th percentile. Discharge tends to be accumulating later in the year across much of the study area.

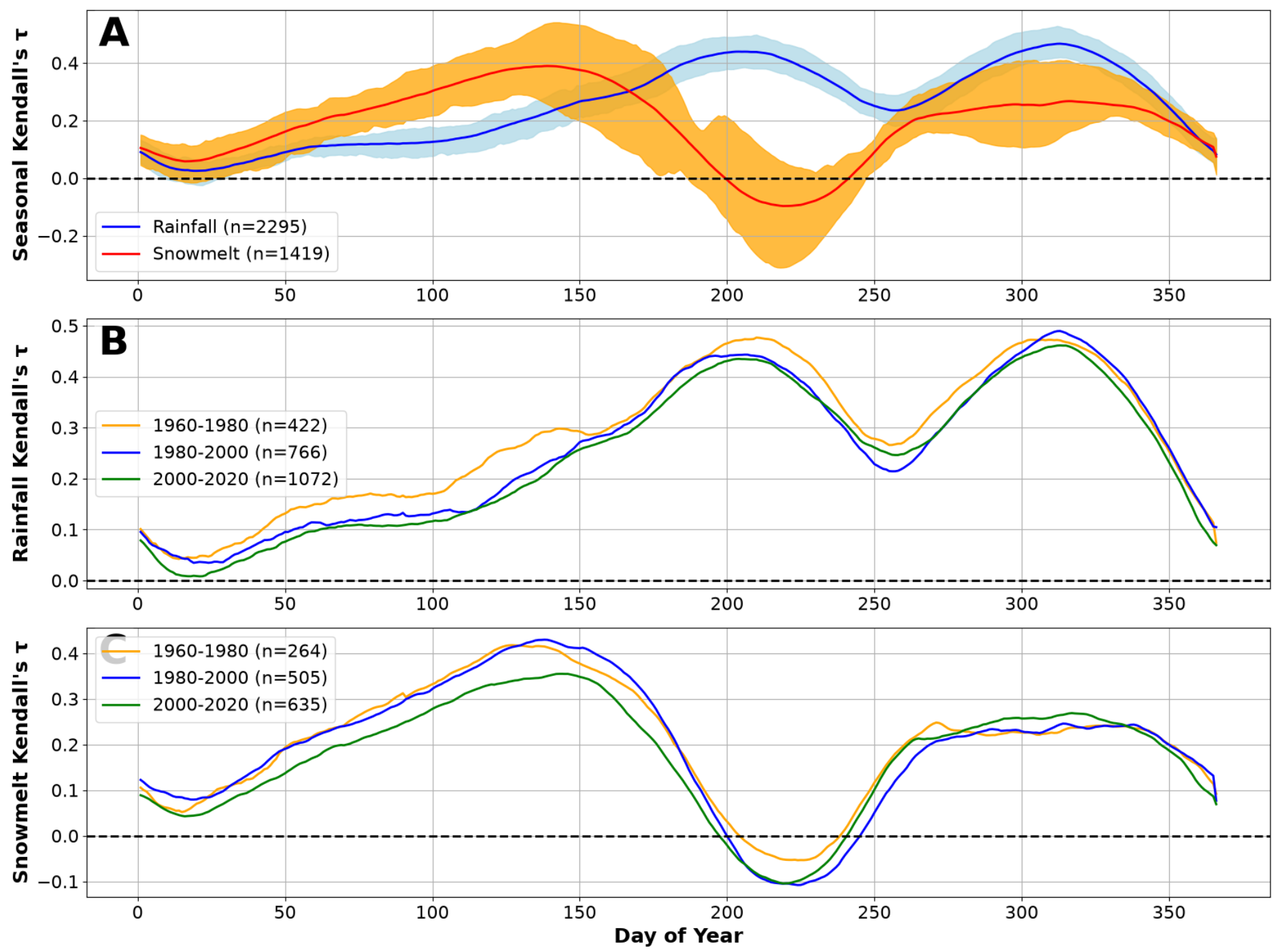

Figure S7: **Seasonal Correlation.** (A) Seasonal (90-day rolling) Kendall's τ statistics for rainfall (blue) and snowmelt (orange) correlated with discharge. Averaged by day-of-year and over all watersheds and all station-years; the number of station-years is summed in the legend. (B) Rainfall and (C) snowmelt Kendall's τ split by decade, showing generally similar seasonal correlation structures through time. Discharge data processed to remove annual baseflow.

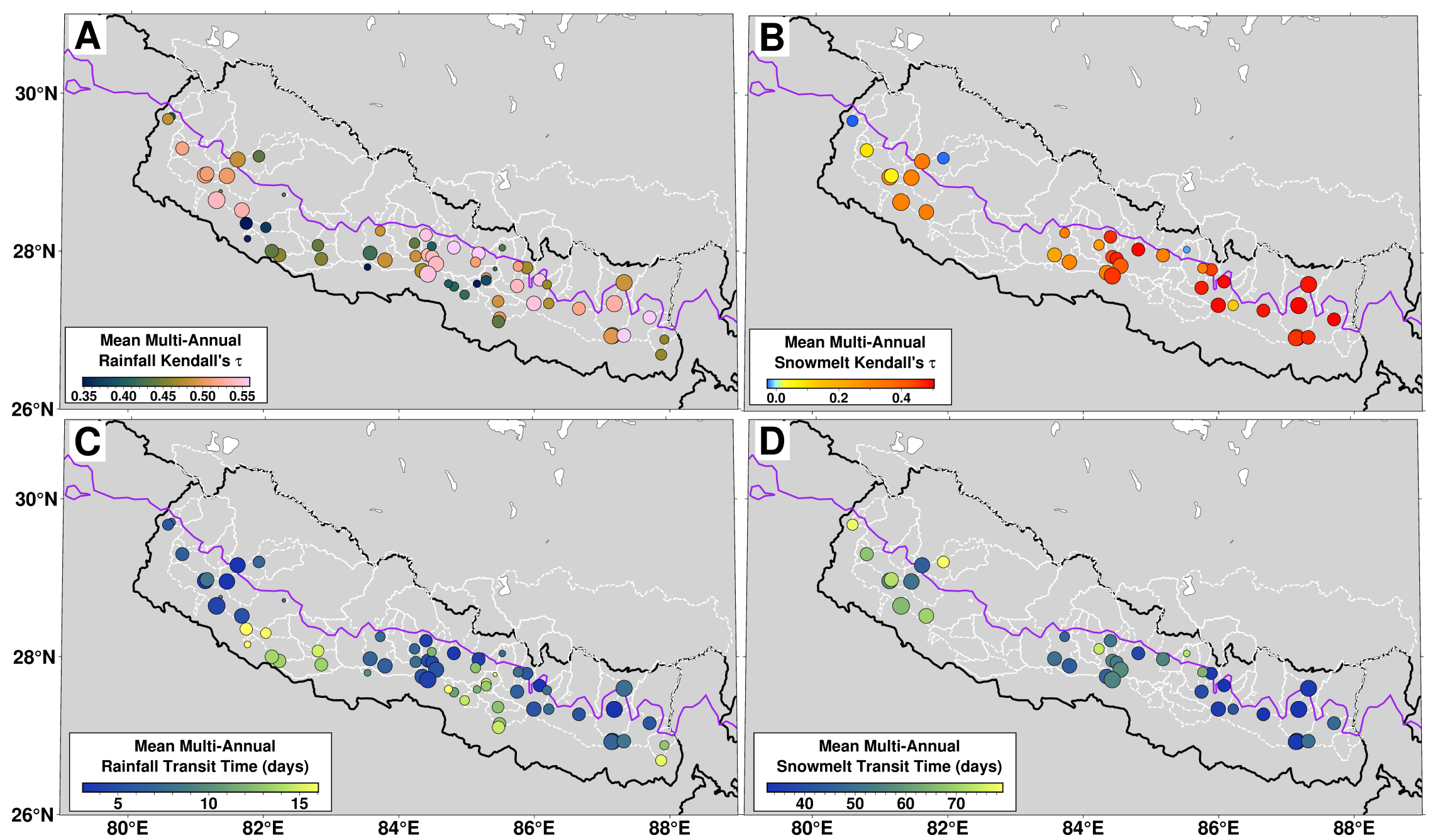

Figure S8: **Inter-Annual correlation.** Kendall's τ averages (three-year window) for (A) rainfall and (B) snowmelt. 2000 m elevation contour shown as purple line. Dots sized by catchment drainage area. Average transit times (three-year window) for (C) rainfall and (D) snowmelt. Discharge data processed to remove annual baseflow.

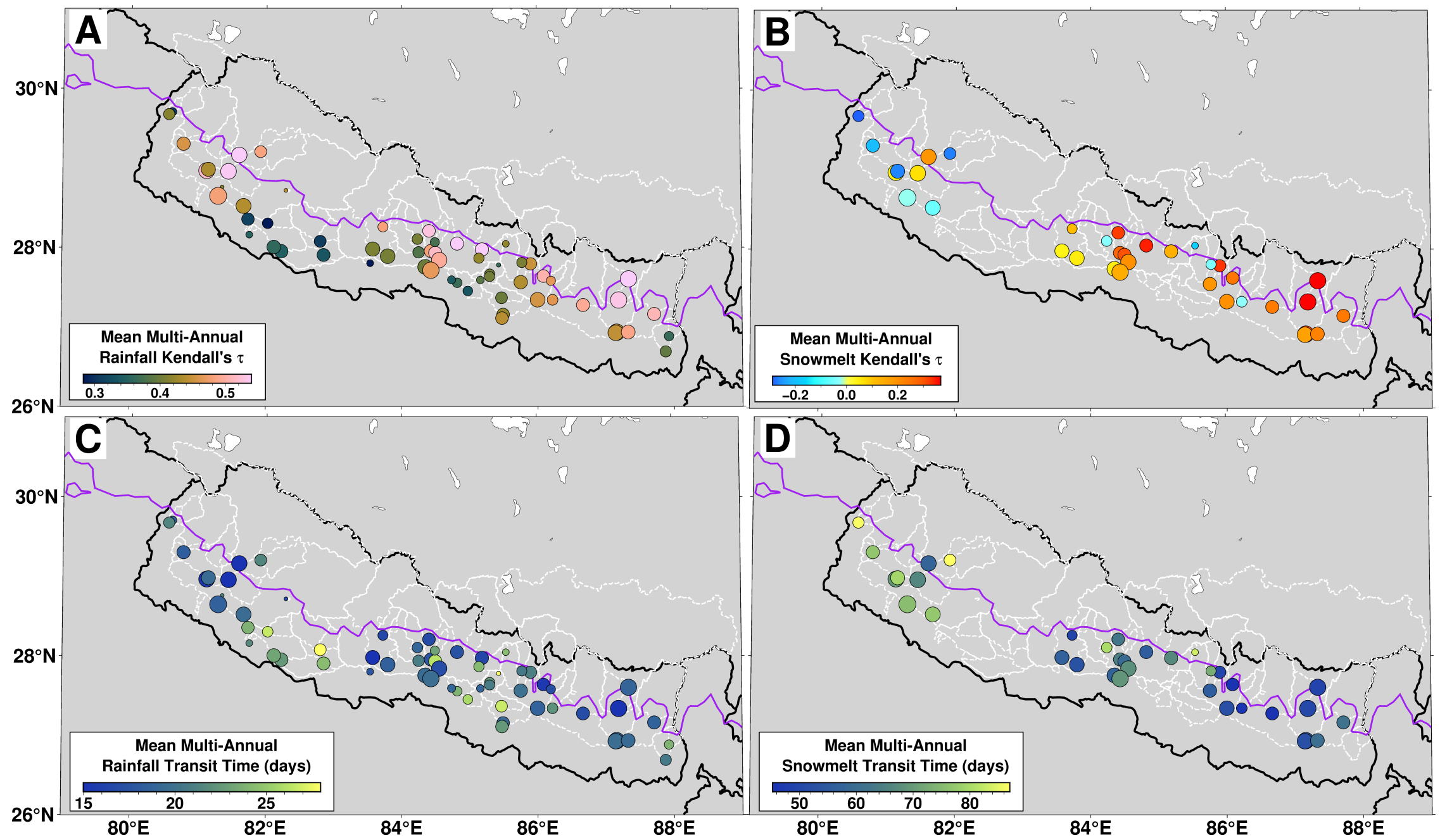

Figure S9: **Inter-Annual correlation.** Kendall's τ averages (three-year window) for (A) rainfall and (B) snowmelt. 2000 m elevation contour shown as purple line. Dots sized by catchment drainage area. Average transit times (three-year window) for (C) rainfall and (D) snowmelt. Discharge data processed to correct rating-curve shifts using BEAST.

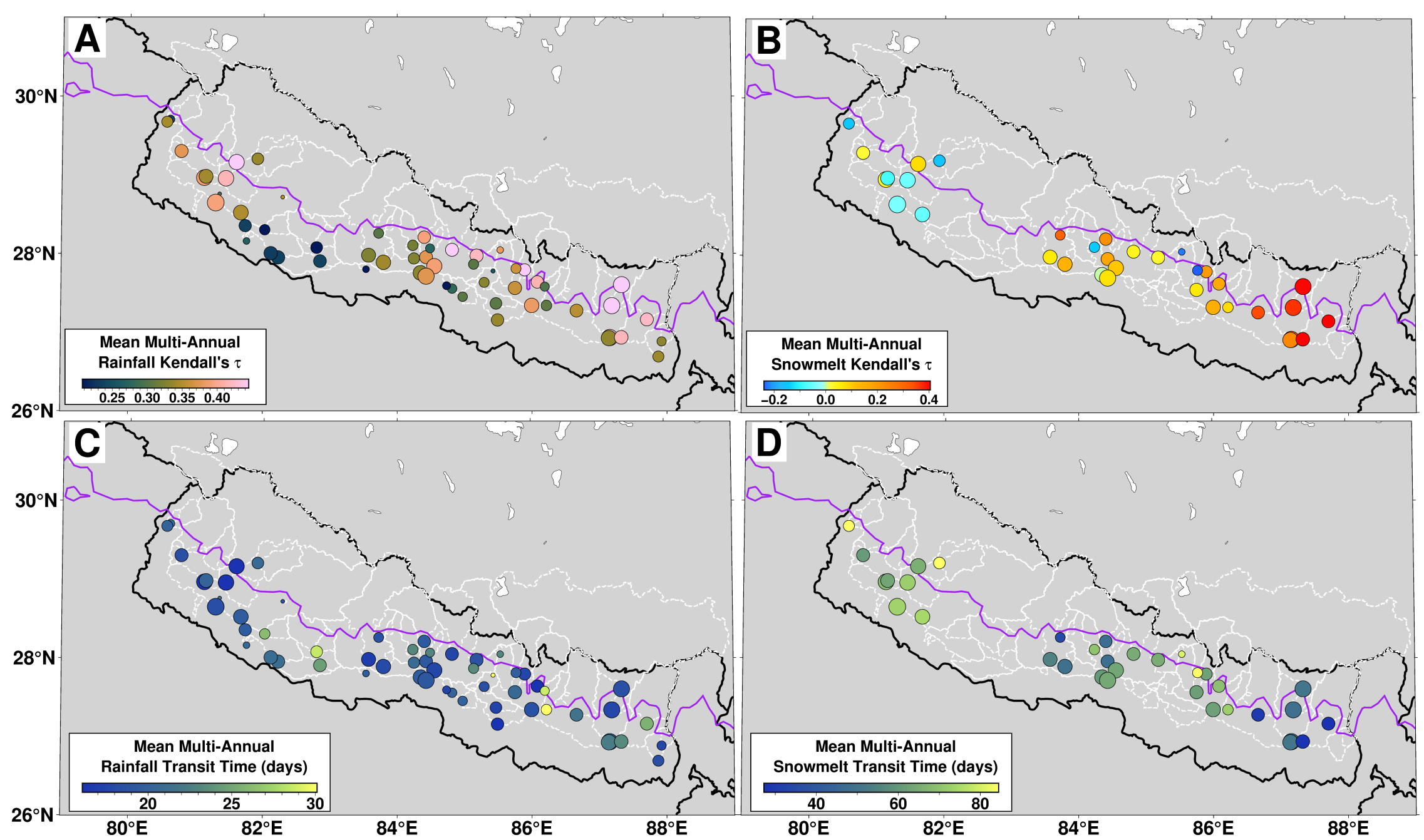

Figure S10: **Inter-Annual correlation.** Kendall's τ averages (three-year window) for (A) rainfall and (B) snowmelt. 2000 m elevation contour shown as purple line. Dots sized by catchment drainage area. Average transit times (three-year window) for (C) rainfall and (D) snowmelt. Discharge data compared to GLDAS instead of ERA5 data.

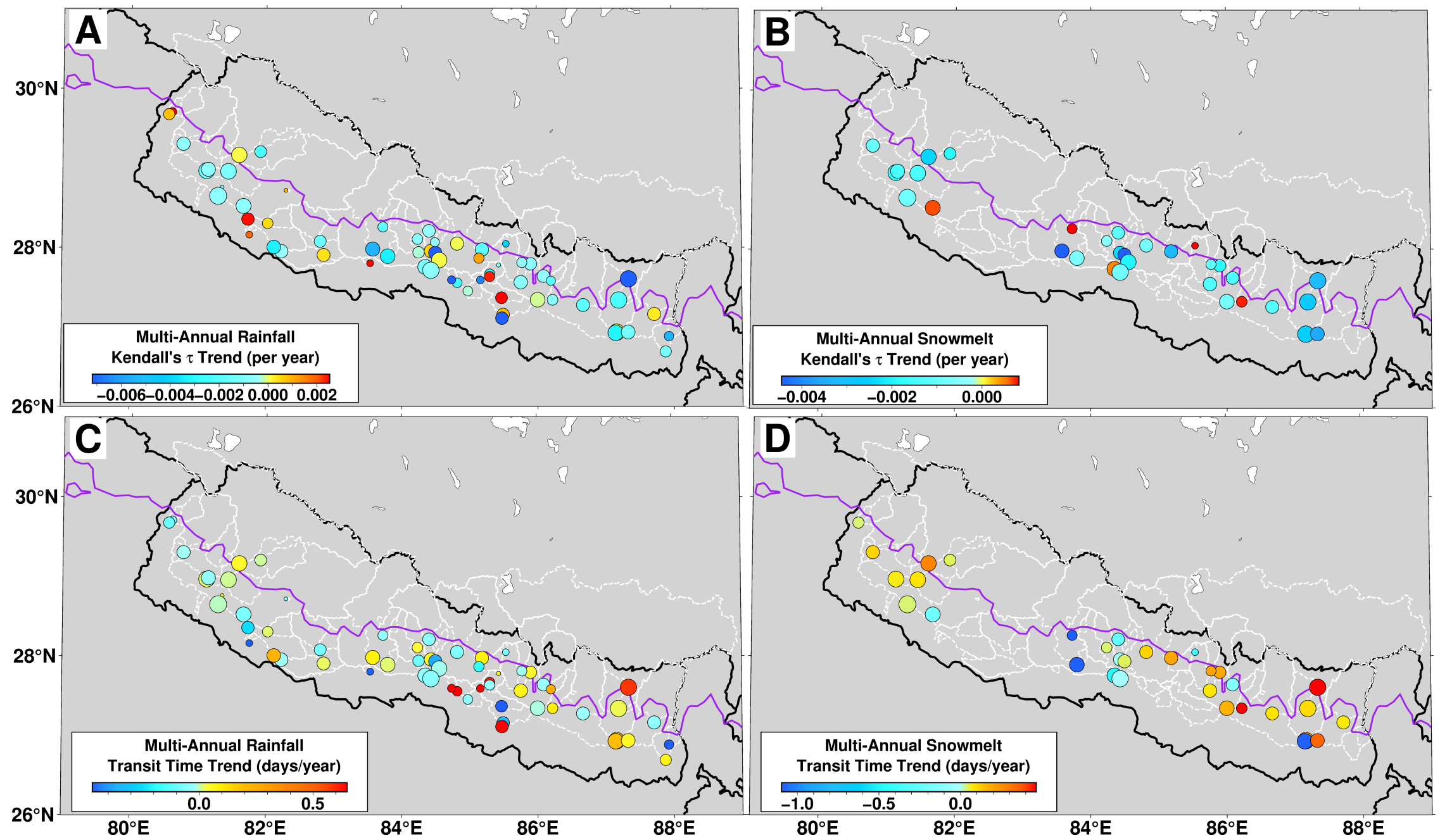

Figure S11: **Precipitation-Discharge Correlation Changes**. Kendall's τ trend between rainfall and discharge (A) and snowmelt and discharge (B). 2000 m elevation contour shown as purple line. Dots sized by catchment drainage area. Trend in transit time for (C) rainfall and (D) snowmelt. Discharge data processed to correct rating-curve shifts using BEAST.

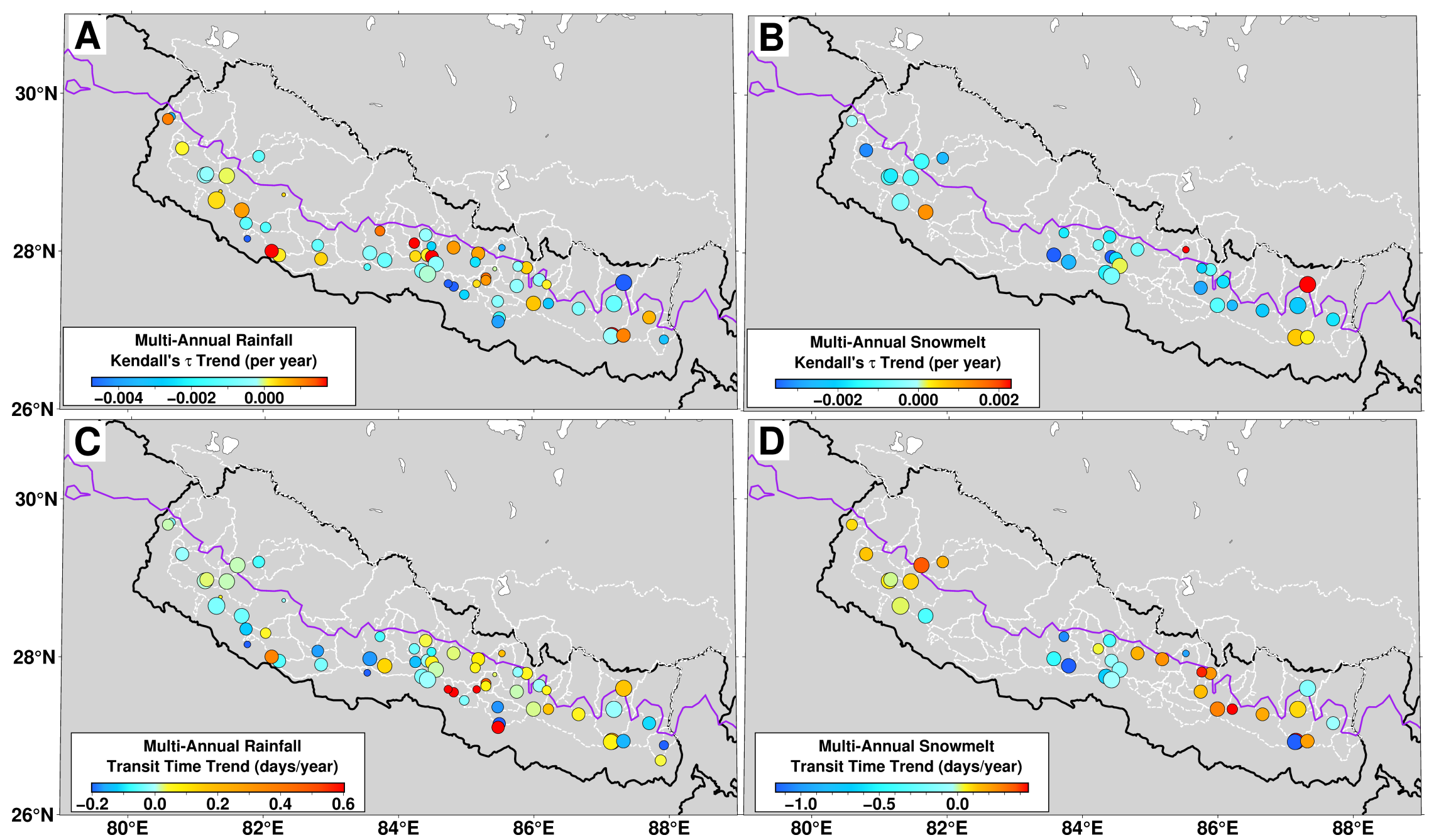

Figure S12: **Precipitation-Discharge Correlation Changes**. Kendall's τ trend between rainfall and discharge (A) and snowmelt and discharge (B). 2000 m elevation contour shown as purple line. Dots sized by catchment drainage area. Trend in transit time for (C) rainfall and (D) snowmelt. Discharge data processed to remove annual baseflow.

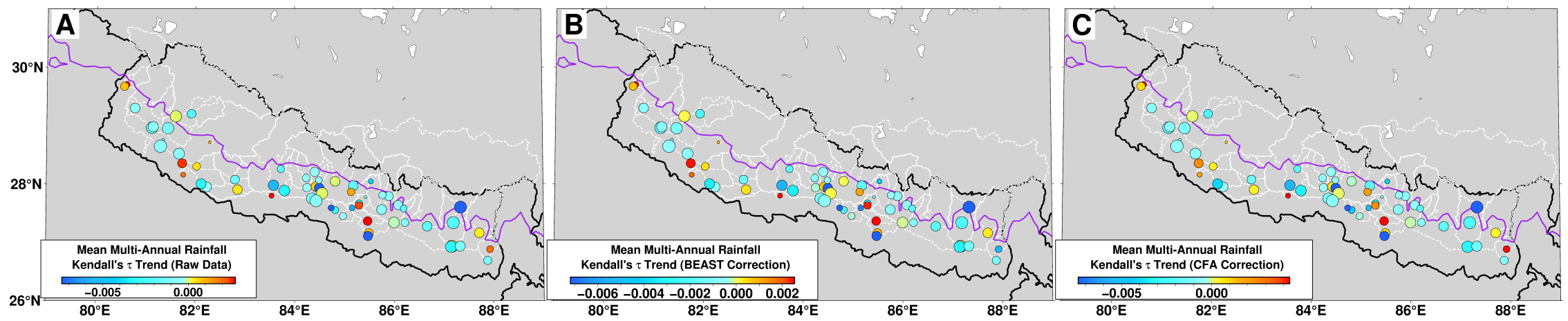

Figure S13: **Precipitation-Discharge Correlation Changes**. Kendall's τ trend between rainfall and discharge. 2000 m elevation contour shown as purple line. Dots sized by catchment drainage area. (A) Raw data, (B) BEAST correction, (C) CFA correction.

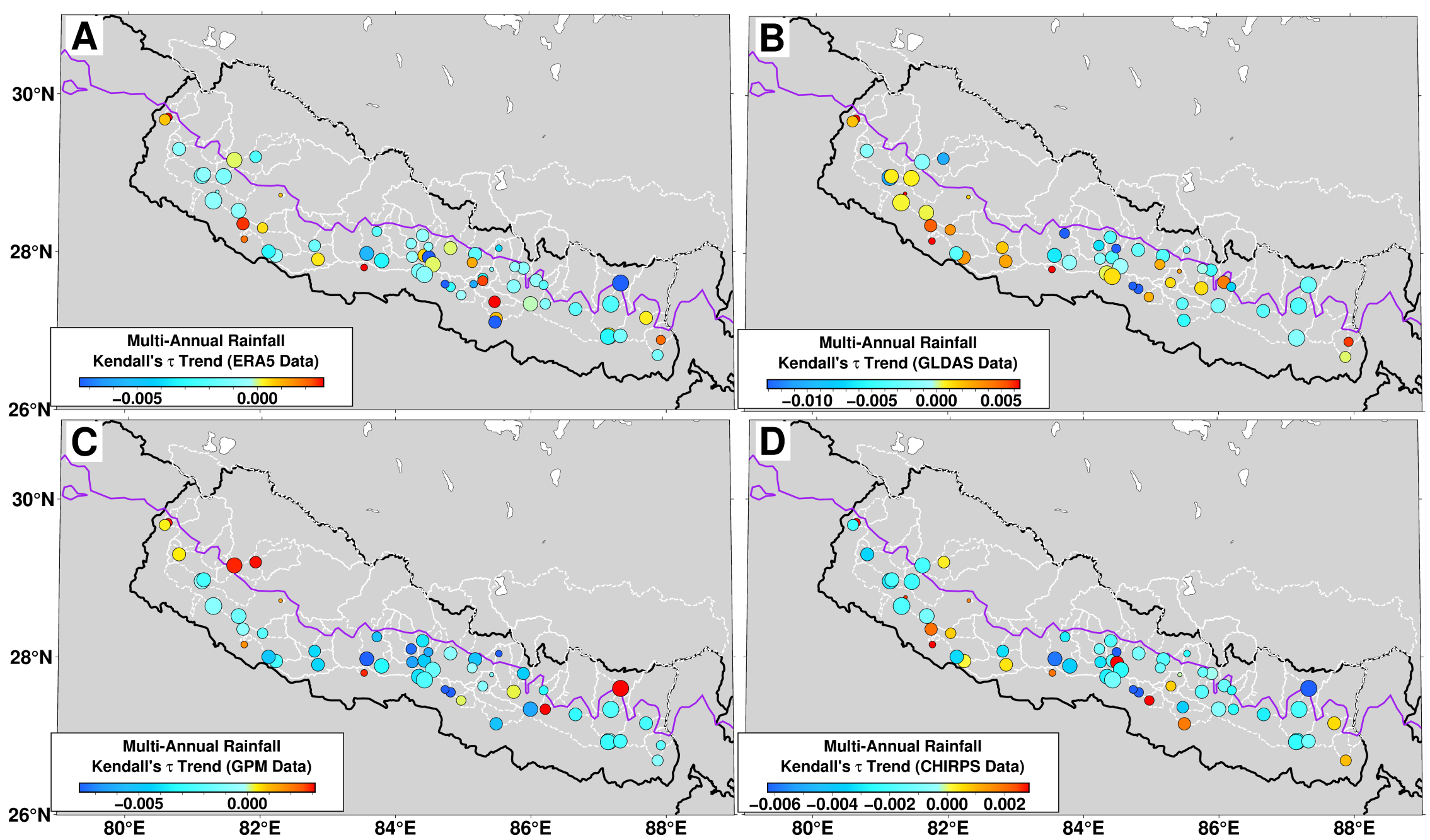

Figure S14: **Precipitation-Discharge Correlation Changes**. Kendall's τ trend between rainfall and discharge. 2000 m elevation contour shown as purple line. Dots sized by catchment drainage area. (A) ERA5 data, (B) GLDAS data, (C) GPM data, (D) CHIRPS data.

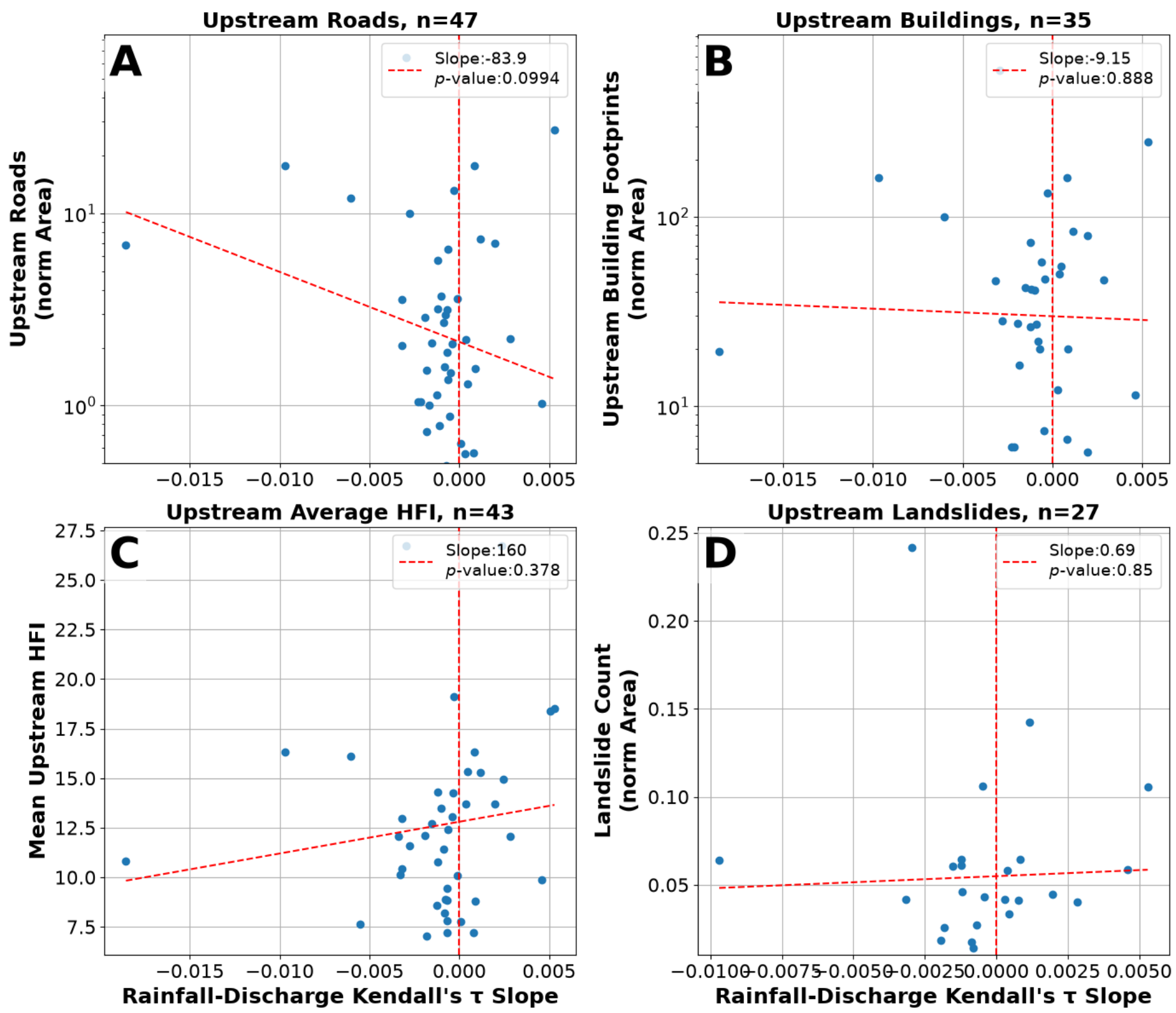

Figure S15: **Impact of Human Infrastructure**. Comparison of Rainfall-Discharge Kendall's τ trends to upstream infrastructure (A: roads, B: buildings, C: Human Footprint Index) and (D) landslide frequency. The relationships between landscape modification and correlation trends are generally weak.

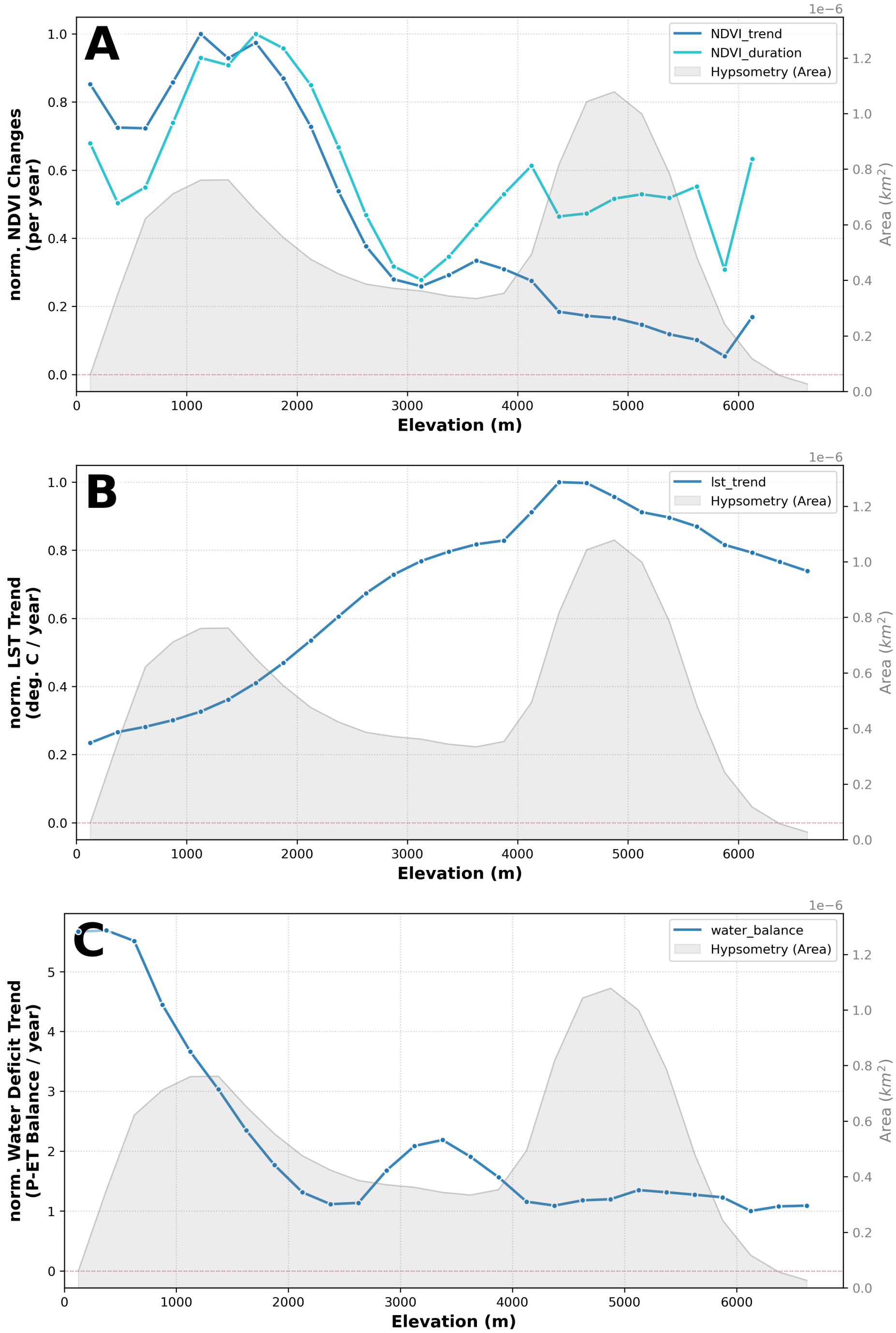

Figure S16: **Role of Hypsometry in Environmental Trends**. Comparison of (A) NDVI, (B) LST, and (C) Water Balance trends to hypsometry (250 m elevation bins). Each binned average line normalized to the maximum to show relative differences. Low elevations tend to have lower LST trends but stronger NDVI and water balance changes.

Table S3: Summary statistics across the four quadrants (ref. Figure 6) for Raw Discharge-Rainfall (1950–2025).

Variable	UL (Faster/ Stronger)	UR (Slower/ Stronger)	LL (Faster/ Decorrel.)	LR (Slower/ Decorrel.)
Num. Stations	14	6	17	26
**Plotted Phase-Space Coordinates**				
Transit Time Trend (x-axis)	-0.41 ± 0.61	0.07 ± 0.06	-0.04 ± 0.03	0.27 ± 0.33
Coupling Trend (Kendall's τ, y-axis)	0.002 ± 0.002	0.001 ± 0.002	-0.002 ± 0.003	-0.003 ± 0.004
**Other Drivers / Trends**				
Baseflow Index Trend (BFI/year)	-0.001 ± 0.003	$-2.5\times10^{-5}\pm1.3\times10^{-3}$	$-3.8\times10^{-4}\pm8.6\times10^{-4}$	$-3.8\times10^{-4}\pm2.6\times10^{-3}$
Heavy Precip. Trend (R95p/year)	0.002 ± 0.004	0.004 ± 0.005	$4.1\times10^{-5}\pm4.6\times10^{-3}$	$8.0\times10^{-4}\pm2.7\times10^{-3}$
Precip. Intensity Trend (SDII, mm/day/year)	$3.5\times10^{-5}\pm7.7\times10^{-5}$	$3.3\times10^{-5}\pm5.8\times10^{-5}$	$2.0\times10^{-7}\pm3.8\times10^{-5}$	$-2.1\times10^{-6}\pm7.7\times10^{-5}$
Total Evaporation Trend (ET, m/year)	-0.002 ± 0.005	$-5.3\times10^{-4}\pm1.7\times10^{-3}$	$1.1\times10^{-4}\pm3.1\times10^{-3}$	$-7.2\times10^{-4}\pm5.7\times10^{-3}$
Available Water Trend ($P-\mathrm{ET}$, m/year)	0.005 ± 0.016	0.003 ± 0.003	$2.2\times10^{-4}\pm1.3\times10^{-2}$	-0.001 ± 0.017
LST Trend (°C/year)	0.010 ± 0.005	0.01 ± 0.01	0.01 ± 0.00	0.01 ± 0.01
NDVI Trend (NDVI/year)	0.002 ± 0.001	0.002 ± 0.001	0.002 ± 0.001	0.002 ± 0.001

Table S4: Summary statistics across the four quadrants for Quickflow-Rainfall (ref. Figure 6) (1950–2025).

Variable	UL (Faster/ Stronger)	UR (Slower/ Stronger)	LL (Faster/ Decorrel.)	LR (Slower/ Decorrel.)
Num. Stations	13	14	18	18
**Plotted Phase-Space Coordinates**				
Transit Time Trend (x-axis)	-0.06 ± 0.05	0.22 ± 0.38	-0.29 ± 0.59	0.24 ± 0.37
Coupling Trend (Kendall's τ, y-axis)	$6.8 \times 10^{-4} \pm 5.6 \times 10^{-4}$	0.001 ± 0.001	-0.002 ± 0.003	-0.002 ± 0.003
**Other Drivers / Trends**				
Baseflow Index Trend (BFI/year)	$-4.4 \times 10^{-4} \pm 1.1 \times 10^{-3}$	$-2.3 \times 10^{-5} \pm 1.1 \times 10^{-3}$	$-6.3 \times 10^{-4} \pm 3.0 \times 10^{-3}$	-0.002 ± 0.003
Heavy Precip. Trend (R95p/year)	0.002 ± 0.002	$-1.9 \times 10^{-5} \pm 5.0 \times 10^{-3}$	0.002 ± 0.005	0.001 ± 0.003
Precip. Intensity Trend (SDII, mm/day/year)	$3.2 \times 10^{-5} \pm 3.8 \times 10^{-5}$	$-2.3 \times 10^{-5} \pm 9.0 \times 10^{-5}$	$1.8 \times 10^{-5} \pm 6.8 \times 10^{-5}$	$2.0 \times 10^{-5} \pm 6.5 \times 10^{-5}$
Total Evaporation Trend (ET, m/year)	0.001 ± 0.003	$-2.5 \times 10^{-4} \pm 1.5 \times 10^{-3}$	-0.001 ± 0.004	-0.001 ± 0.007
Available Water Trend ($P - \mathrm{ET}$, m/year)	0.01 ± 0.01	-0.005 ± 0.013	-0.001 ± 0.011	0.002 ± 0.019
LST Trend (°C/year)	0.01 ± 0.00	0.01 ± 0.00	0.01 ± 0.01	0.01 ± 0.00
NDVI Trend (NDVI/year)	0.002 ± 0.001	0.001 ± 0.001	0.002 ± 0.001	0.002 ± 0.001
